# More than Depression: Doctoral Students’ Mental Health in China: A Systematic Review and Meta-Analysis

**DOI:** 10.3390/bs16081355

**Published:** 2026-08-06

**Authors:** Huabing Liu, Yanmin Gao, Nayila Tuerxun, Latifat Cabirou

**Affiliations:** 1 School of Education, Shanghai Jiao Tong University, Shanghai 200240, China; hygge_1127@sjtu.edu.cn (Y.G.); nayilatuerxun@sjtu.edu.cn (N.T.); 2 Department of Special Education and Counseling, College of Education, Auburn University, Auburn, AL 36849, USA; loc0005@auburn.edu

**Keywords:** doctoral students, mental health, interpersonal sensitivity, content analysis, meta-analysis, SCL-90

## Abstract

The mental health of doctoral students is a critical concern in higher education. Despite growing research attention, Chinese-language scholarship remains underrepresented in international academic discourse because of language barriers. This study systematically reviewed Chinese-language empirical research on doctoral students’ mental health to summarize current findings and provide a comprehensive account of research trends. Following PRISMA guidelines, we searched three major Chinese databases (CNKI, cqVIP, and Wanfang Data) from inception through December 2023. A content analysis examined research characteristics, methods, and findings across 35 eligible studies. A meta-analysis synthesized SCL-90 data from five studies comparing doctoral students with master’s students, Chinese population norms, and Chinese youth norms using random-effects models. The content analysis revealed a three-stage evolution of the field: emergence (2005–2011), consolidation (2012–2017), and diversification (2018–2023), with increasing methodological sophistication and attention to vulnerable subgroups. The meta-analysis clarified inconsistent findings from individual studies, showing small differences between doctoral and master’s students but more widespread symptom elevations relative to Chinese population and youth norms. Interpersonal sensitivity showed the most consistent elevation across all comparisons, particularly relative to youth norms, followed by depression and somatization. Implications for practice, research gaps, and directions for future research are discussed.

## 1. Introduction

The mental health and psychological well-being of doctoral students have emerged as critical concerns in higher education worldwide. Large-scale studies across multiple countries have consistently documented substantial mental health challenges among students pursuing doctoral degrees. For instance, Nature’s international survey of more than 6300 doctoral students across disciplines found that 36% of respondents had sought help for anxiety or depression related to their doctoral studies, with work–life balance and research setbacks identified as primary stressors (Woolston, 2019). Similarly, a survey of 2279 graduate students across 26 countries found that approximately 40% experienced moderate to severe anxiety or depression, with doctoral students reporting rates six times higher than those in the general population (Evans et al., 2018). Together, these findings demonstrate the pervasive nature of mental health challenges in doctoral education.

The mental health challenges faced by Chinese doctoral students warrant particular attention. Research has indicated that their psychological symptom scores were significantly higher than Chinese population norms, with concerning implications for academic success and personal development (Lu et al., 2012). Despite these mental health concerns, the proportion of doctoral students who seek professional psychological services remains low (Hyun et al., 2007). Studies have identified several barriers to help-seeking, including stigma surrounding psychological problems, inadequate social support, and a mismatch between counseling services and students’ expectations (H. Liu et al., 2020; Zeng & Zhang, 2023). The prevalence and often concealed nature of these concerns may adversely affect Chinese doctoral students’ academic progress, personal development, and psychological health, potentially undermining China’s efforts to cultivate innovative, highly skilled researchers (S. Guo, 2023). Poor mental health among doctoral students can impair academic performance, diminish academic aspirations (Q. Wang & Jiang, 2023), and contribute to program interruption or early termination (Wollast et al., 2018). Furthermore, doctoral students experiencing emotional distress may be more likely to encounter interpersonal conflict and feelings of frustration or powerlessness in social interactions (Lau & Pretorius, 2019). Together, these academic and social challenges can create a cycle of stress that compromises the quality of doctoral education and the development of future academic talent.

### 1.1. Mental Health Studies in Chinese Doctoral Students

Over the past two decades, China has experienced substantial growth in doctoral education, with enrollment rising from approximately 68,370 in 2012 to 138,951 in 2022 (Ministry of Education of the People’s Republic of China, 2024). This expansion has positioned China as home to the world’s largest higher education system (H. Wu et al., 2024), with the country surpassing the United States in annual doctoral graduates in 2019. Alongside this quantitative growth, China has implemented quality assurance initiatives and reforms in doctoral education. These measures include establishing a comprehensive quality assurance system for graduate education, implementing an “application-review” procedure for doctoral admissions, and developing dual-mentor structures that integrate industry, education, and research (Zhu et al., 2024). To sustain a robust doctoral education system, higher education administrators must address risks to students’ mental health. Understanding the factors associated with positive mental health and psychological distress is therefore essential for promoting psychological well-being and strong academic outcomes.

Contemporary research on Chinese doctoral students reflects their experiences across various domains, including career aspirations (Gu et al., 2018), employment choices (W. Sun et al., 2018), creativity development (Cao et al., 2024), environmental factors affecting motivation and research outcomes (S. Huang & Yang, 2024), publication impact (Horta & Li, 2023), and academic identity formation (H. Zhang et al., 2024). This research has identified parallels between Chinese and international doctoral students in areas such as social support needs, student–supervisor relationships, and career adaptation challenges (Xu & Liu, 2023; L. Li et al., 2023). However, these common challenges manifest distinctively within Chinese cultural and institutional contexts. For example, although impostor syndrome and academic pressure affect doctoral students globally (Leick & Köstner, 2025; Sverdlik et al., 2018), their expression in Chinese settings is shaped by cultural concepts such as “face” (mianzi) and a collective achievement orientation (Dai & Elliot, 2023). Similarly, as mentoring relationships are important across contexts (Y. Yang & MacCallum, 2022), traditional Chinese academic hierarchies create distinct dynamics that can influence student well-being (Xu & Liu, 2023; W. Liang et al., 2021).

International reviews have examined doctoral students’ mental health from complementary perspectives. Hazell et al. (2020) synthesized evidence on mental health problems and associated risk and protective factors. Satinsky et al. (2021) estimated the prevalence of depression, anxiety, and suicidal ideation. Jackman et al. (2022) focused on the early stages of doctoral study. Martínez-García et al. (2024) and Y. Guo and Liu (2026) examined broader conceptions, determinants, and interventions related to doctoral well-being. Although these reviews establish the global importance of doctoral students’ mental health, they predominantly synthesize English-language research. Their findings may not fully generalize to China because doctoral training is embedded in distinct cultural and academic systems (Brailovskaia et al., 2018). Consequently, Chinese-language scholarship remains underrepresented in international discussions, limiting understanding of how doctoral students’ mental health has been conceptualized and investigated within China. A review centered on Chinese-language scholarship is therefore needed to determine whether established international patterns are reproduced, qualified, or extended in this context.

### 1.2. Present Study

The present review addresses this gap by bringing a bounded corpus of Chinese-language research into the international literature and examining the development and methodological characteristics of this research tradition. We employ two complementary analytical approaches. First, through content analysis of Chinese-language studies published between 2000 and 2023, we examine the development of the field, including its research objectives, theoretical frameworks, samples, measures, methodological approaches, and principal findings. Second, because the content analysis revealed inconsistent conclusions regarding the severity of doctoral students’ mental health symptoms, we conduct a secondary meta-analysis of studies reporting complete Symptom Checklist-90 data. This analysis compares Chinese doctoral students with master’s students, Chinese population norms, and Chinese youth norms. Together, these approaches enable us to contextualize symptom patterns within the broader development of Chinese research, compare the findings with international evidence, and identify priorities for future research and institutional support.

## 2. Materials and Methods

### 2.1. Eligibility Criteria

Studies were eligible if they (a) reported original empirical data; (b) examined full-time doctoral students who were Chinese nationals and were studying at institutions in mainland China; (c) quantitatively or qualitatively assessed mental health outcomes; (d) were published in Chinese; and (e) appeared in journals listed in the Peking University Core Journals or the Chinese Social Sciences Citation Index. We limited eligibility to the two indexing systems because they provide explicit journal lists, allowing the publication criterion to be applied consistently and defining a transparent and reproducible corpus of Chinese journal scholarship. Indexing status was used as an eligibility boundary rather than as an indicator of study-level methodological quality. We excluded theoretical papers, reviews, commentaries, studies focused primarily on non-doctoral populations, studies that did not report mental health outcomes, non-indexed journal articles, conference proceedings, dissertations, and unpublished research.

### 2.2. Information Sources and Search Strategy

The review followed the Preferred Reporting Items for Systematic Reviews and Meta-Analyses (PRISMA) 2020 reporting guideline (Page et al., 2021). We searched three major Chinese academic databases from inception through 31 December 2023: China National Knowledge Infrastructure (CNKI), China Science and Technology Journal Database (cqVIP), and Wanfang Data. The search combined a population block (“博士生” OR “博士研究生” OR “doctoral student*” OR “PhD student*”) with an outcome block (“心理健康” OR “mental health” OR “心理问题” OR “psychological problem*” OR “心理压力” OR “psychological stress” OR “抑郁” OR “depress*” OR “焦虑” OR “anxiety”) using the Boolean operator AND; terms within each block were combined using OR. After entering the search terms, we applied the journal-source filters available in each database to retain records published in journals included in the Peking University Core Journals or the Chinese Social Sciences Citation Index (CSSCI). Accordingly, the numbers reported at the identification stage represent the records retrieved after these journal-index filters were applied, rather than the total unfiltered search results. The same search concepts were applied in each database.

### 2.3. Selection Process

**Figure 1:**
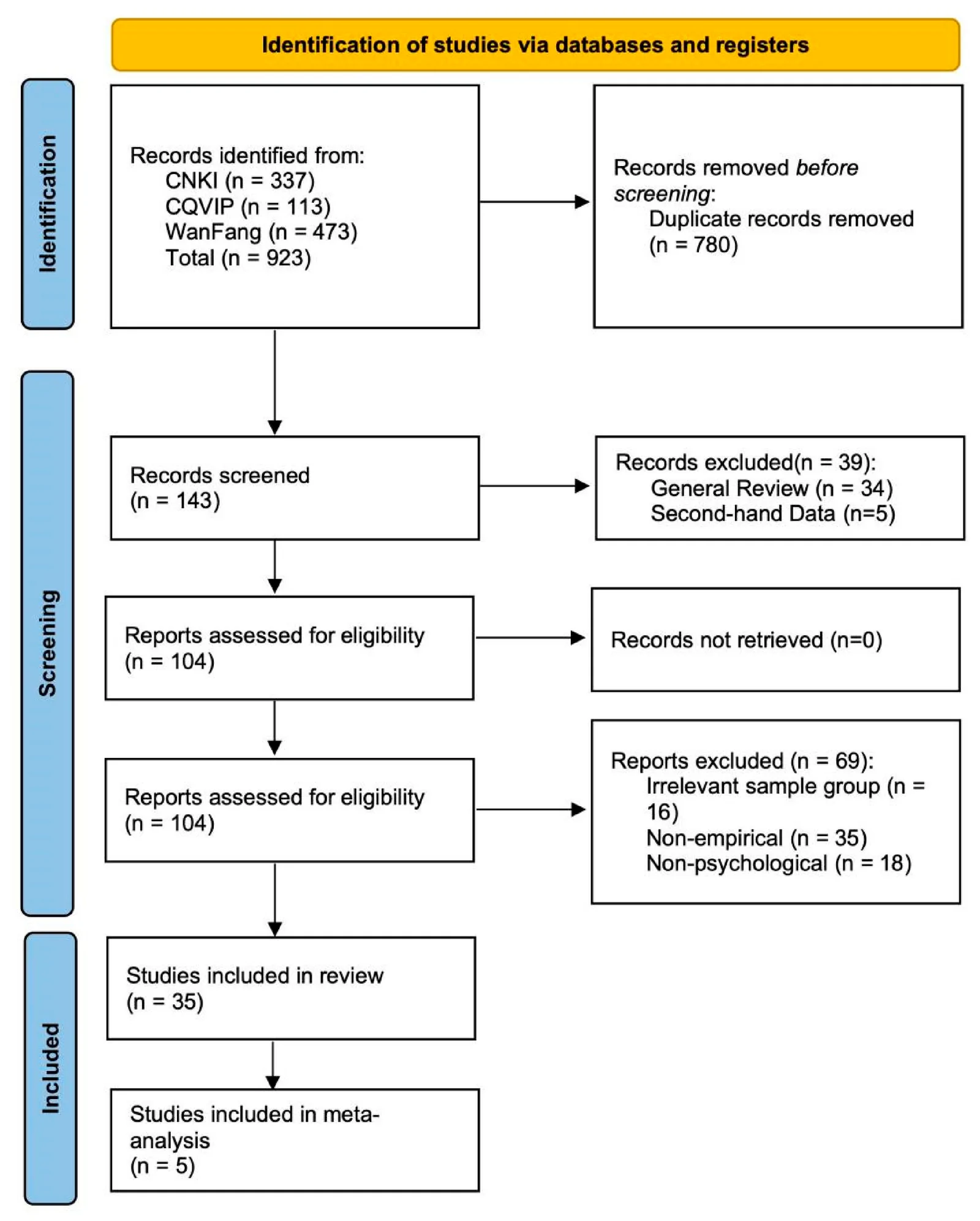
PRISMA flow diagram for identifying and screening eligible studies of doctoral students’ mental health in China.

Three researchers independently screened titles and abstracts using Zotero 6.0.30 reference management software. The full texts of potentially eligible studies were retrieved and independently assessed by the same three researchers against the eligibility criteria. Screening disagreements were resolved through discussion with a fourth researcher. The selection process yielded 35 eligible studies from an initial pool of 923 records for the content analysis. Details of the screening and selection process are provided in Figure 1.

### 2.4. Data Collection Process and Data Items

For the content analysis, the article was the unit of analysis, with research questions, samples, variables or themes, and findings treated as specific subunits. Extracted items covered study characteristics, participant characteristics, publication year, content area, research methods and measures, and study findings. For the meta-analysis, extracted items included sample sizes, group means, standard deviations, scores for all SCL-90 subscales, and within-study comparative statistics (e.g., t values) when reported. Studies without complete SCL-90 summary data for all subscales were not included in the quantitative synthesis.

### 2.5. Content Analysis

We used directed content analysis (Hsieh & Shannon, 2005) to analyze the 35 publications. We first developed and iteratively refined a comprehensive coding framework. Initial categories were derived from commonly used frameworks in the mental health literature. After receiving coding training, three research team members independently coded two representative articles. The team then discussed the results, refined the codebook, and aligned their interpretation of the coding categories. The final codebook was established after all team members reached consensus.

The three researchers then independently coded the remaining 33 studies using the finalized framework (Appendix A Table A1), which covered five domains: study characteristics (e.g., sample size and research methods), participant characteristics (e.g., gender and discipline), publication year, content area (e.g., research topics), and research findings. A variable not mentioned in an article was coded as “Not Reported”; information that was present but insufficient for a definitive judgment was coded as “Unclear”; and a variable that did not apply to the study design was coded as “Not Applicable.” These categories were kept separate from the substantive codes so that incomplete reporting did not distort the descriptive summaries.

Interrater reliability was assessed using Cohen’s kappa (κ = 0.734), indicating substantial agreement (Landis & Koch, 1977). Coding discrepancies were documented and resolved through team discussion until consensus was reached. Data were analyzed using SPSS version 26.0, with descriptive statistics used to characterize the distribution and frequency of coded elements across all domains.

### 2.6. Meta-Analysis Eligibility, Effect Measures, and Synthesis Methods

Through the content analysis, we identified inconsistent findings concerning the severity of doctoral students’ mental health symptoms. Because the Chinese version of the Symptom Checklist-90 (SCL-90; Z. Wang, 1984) was the most commonly used measure of symptom severity, we selected studies that provided complete and comparable SCL-90 data, including means, standard deviations, and scores for all subscales. Pooling these studies allowed us to examine the direction and magnitude of symptom differences more systematically than was possible through narrative comparison alone. Five studies met the inclusion criteria (Table 1), with sample sizes ranging from 191 to 1950 participants. We conducted three comparisons: (1) Chinese doctoral students versus master’s students (*k* = 3 studies), (2) Chinese doctoral students versus Chinese population norms (*k* = 5), and (3) Chinese doctoral students versus Chinese youth norms (*k* = 5). Data for master’s students were extracted from the same studies as the doctoral student data, using comparative statistics (e.g., t values) reported in each study. The Chinese population norms represent standardized SCL-90 scores from a broad adult sample, whereas the youth norms (ages 19–29 years) provide a more developmentally comparable reference group for doctoral students. Both sets of normative data were obtained from Jin et al. (1986), based on 1388 participants.

Although only five studies were eligible, a mini-meta-analysis can provide a useful quantitative summary of effect-size patterns across a small evidence base when its findings are interpreted cautiously (Goh et al., 2016).

Heterogeneity was evaluated using I^2^, τ^2^, and τ. I^2^ describes the proportion of observed variation attributable to between-study heterogeneity; τ^2^ estimates the between-study variance, and τ is the corresponding between-study standard deviation. These complementary statistics were considered alongside differences in study designs, participant samples, and measurement or analytical methods. All analyses were conducted using Comprehensive Meta-Analysis Version 3 with α = 0.05 (two-tailed).

Potential publication bias was assessed using the fail-safe N method and funnel plots. Funnel plots for the available symptom dimensions are provided in Supplementary SA (doctoral-versus-master’s comparisons), Supplementary SB (doctoral-versus-Chinese-population-norm comparisons), and Supplementary SC (doctoral-versus-Chinese-youth-norm comparisons). Egger’s regression test was not conducted because each comparison included no more than five studies, below the commonly recommended minimum of 10 studies for a meaningful regression-based assessment (Sterne et al., 2011). Accordingly, the funnel plots were interpreted descriptively and cautiously rather than as definitive evidence of publication bias.

### 2.7. Risk of Bias and Methodological Reporting Assessment

Two researchers independently assessed the risk of bias in the five analytical cross-sectional studies included in the meta-analysis using the revised JBI critical appraisal tool for analytical cross-sectional studies (Barker et al., 2026). The tool comprises eight questions addressing sample inclusion criteria; measurement of the condition, exposure, and outcomes; identification and management of confounding; statistical analysis; and description of participants and settings. Each item was rated Yes, No, Unclear, or Not Applicable. Disagreements were resolved through discussion until consensus was reached. Overall judgments of low risk, some concerns, or high risk were based on the pattern and importance of the item-level judgments rather than on a numerical cutoff.

For the 35 studies included only in the content analysis, we summarized methodological and reporting characteristics rather than applying a single formal risk-of-bias tool. This approach was used because the corpus combined quantitative, qualitative, and mixed-methods designs and was intended to map the field rather than generate pooled estimates. The coded characteristics included research design, sampling method, participant information, measures, psychometric reporting, and data-analysis methods (Appendix A Table A5).

### 2.8. Protocol and Registration

A protocol was developed before the review commenced; however, the review was not registered.

## 3. Results

### 3.1. Study Selection

The database searches identified 923 records: 337 from CNKI, 113 from cqVIP, and 473 from Wanfang Data. After 780 duplicate records were removed, 143 records underwent title and abstract screening. Thirty-nine records were excluded at this stage (34 reviews and five studies based on secondary data). The remaining 104 reports were assessed in full text, of which 69 were excluded because the population was not relevant (*k* = 16), the report was non-empirical (*k* = 35), or the outcome was not psychological (*k* = 18). Thirty-five publications were included in the content analysis, and five of these provided complete SCL-90 data for the meta-analysis (Figure 1).

### 3.2. Study Characteristics and Publication Trends

#### 3.2.1. Researchers’ Academic Profiles

**Figure A1:**
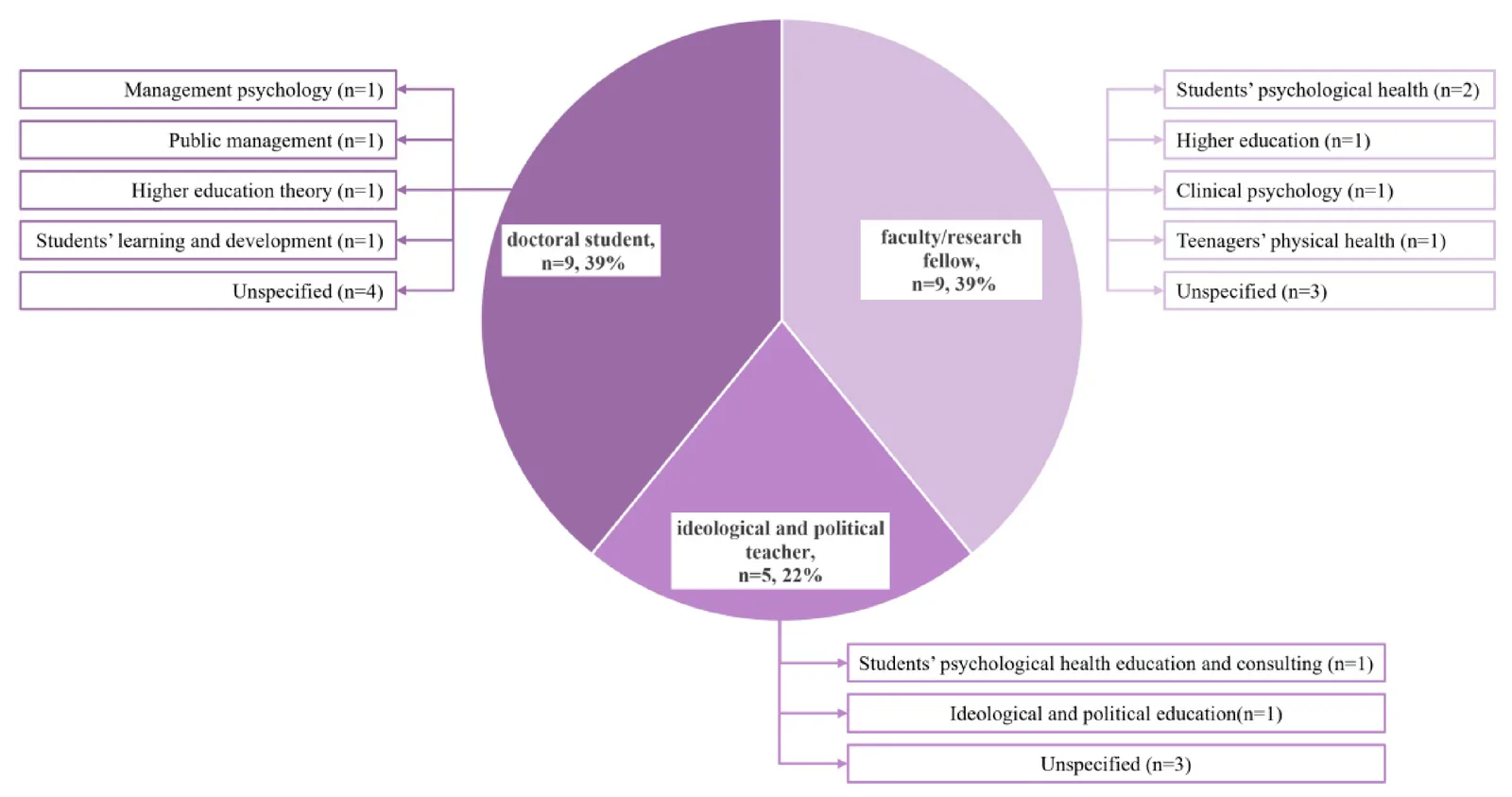
Percentage and frequency of researcher status and research interests.

First authors were commonly full-time teachers or researchers (*k* = 9) or doctoral students (*k* = 9) (Appendix A Figure A1). Among the faculty-led publications, five were authored by researchers whose primary discipline was civics and politics. Publications led by full-time faculty or researchers primarily focused on college student mental health, mental health education and counseling, higher education, and clinical psychology. Doctoral student-led publications focused on the theoretical foundations of higher education, theories of public administration, and college student learning and development.

#### 3.2.2. Publication Year

**Figure 2:**
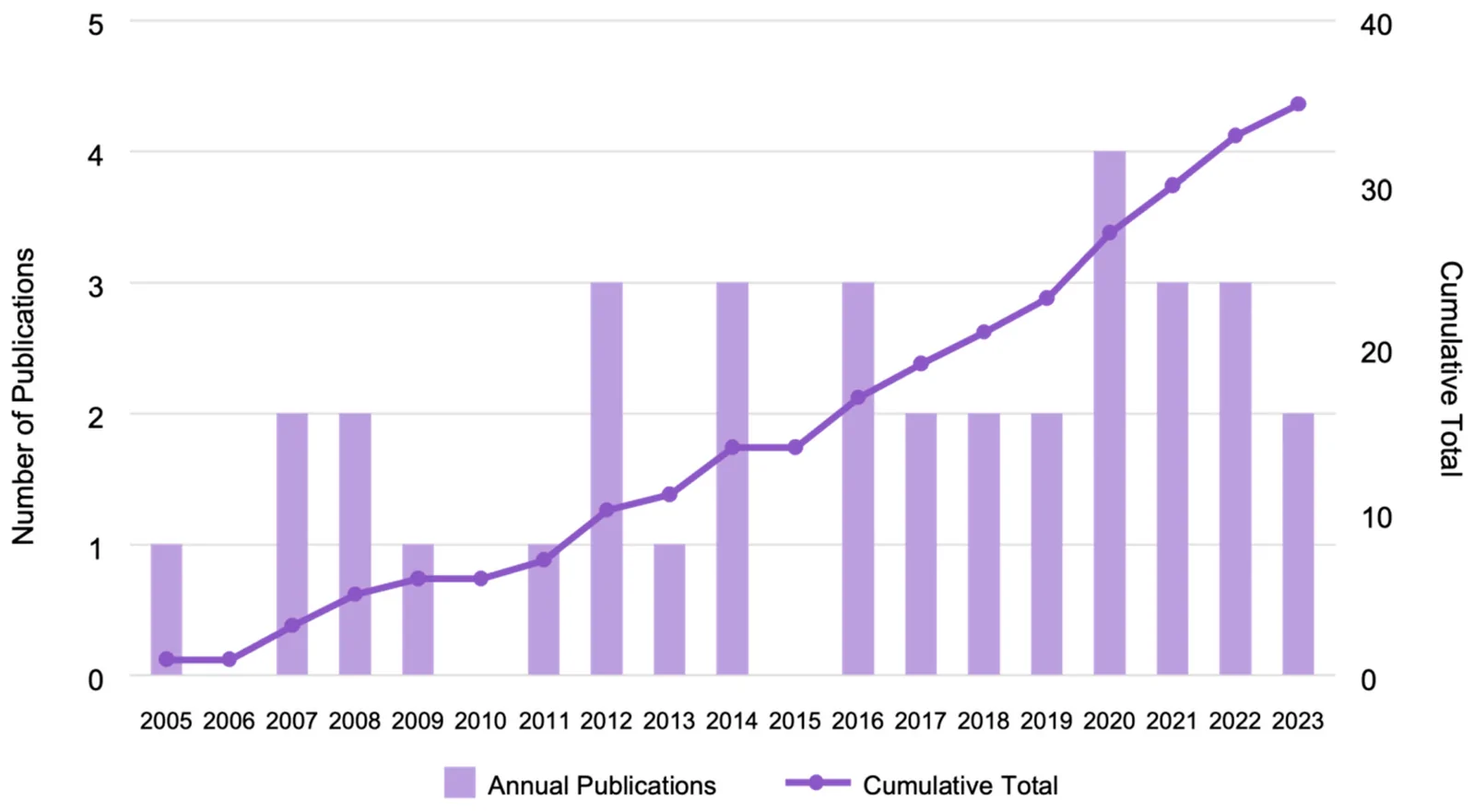
Distribution of publications by year.

The distribution of publications by year, illustrated in Figure 2, revealed a clear developmental trajectory. Empirical research on doctoral students’ mental health emerged in 2005 and showed an overall upward trend, with the number of publications fluctuating from year to year.

### 3.3. Research Designs, Methods, and Measures

#### 3.3.1. Research Topic and Objectives

A total of 65 research questions were investigated across the 35 publications. The most common questions concerned doctoral students’ overall mental health status (*k* = 10), the prevalence of psychological stress (*k* = 5), predictors of mental health (*k* = 5), and anxiety symptoms (*k* = 3).

Following Blaikie’s (2000, p. 72) framework, we classified research objectives as exploratory (investigating new or unfamiliar topics), descriptive (documenting what exists or occurs), explanatory (identifying why phenomena occur through causal relationships), or understanding (interpreting meanings and experiences). Using this classification, the objectives were primarily explanatory (*k* = 16), followed by descriptive (*k* = 10) and understanding (*k* = 8). Examples are provided in Table 2.

#### 3.3.2. Research Designs and Theoretical Frameworks

Of the 35 papers, 26 used a quantitative design, seven used a qualitative design, and two used a mixed-methods design. Surveys (*k* = 25) were the most prevalent quantitative method, whereas interviews, including individual and focus-group interviews (*k* = 7), were the most commonly used qualitative method.

Five of the 35 papers described the specific theoretical foundations or models underpinning their studies. The two quantitative studies used positive psychology theory (Song et al., 2019) and burnout theory (Y. C. Li, 2020). The three qualitative studies drew on marginal theory (Hong et al., 2020), the ailment narrative (Cheng & Li, 2022), and the role-identity interaction model (X. H. Sun & Li, 2023).

#### 3.3.3. Variables and Themes

**Figure 3:**
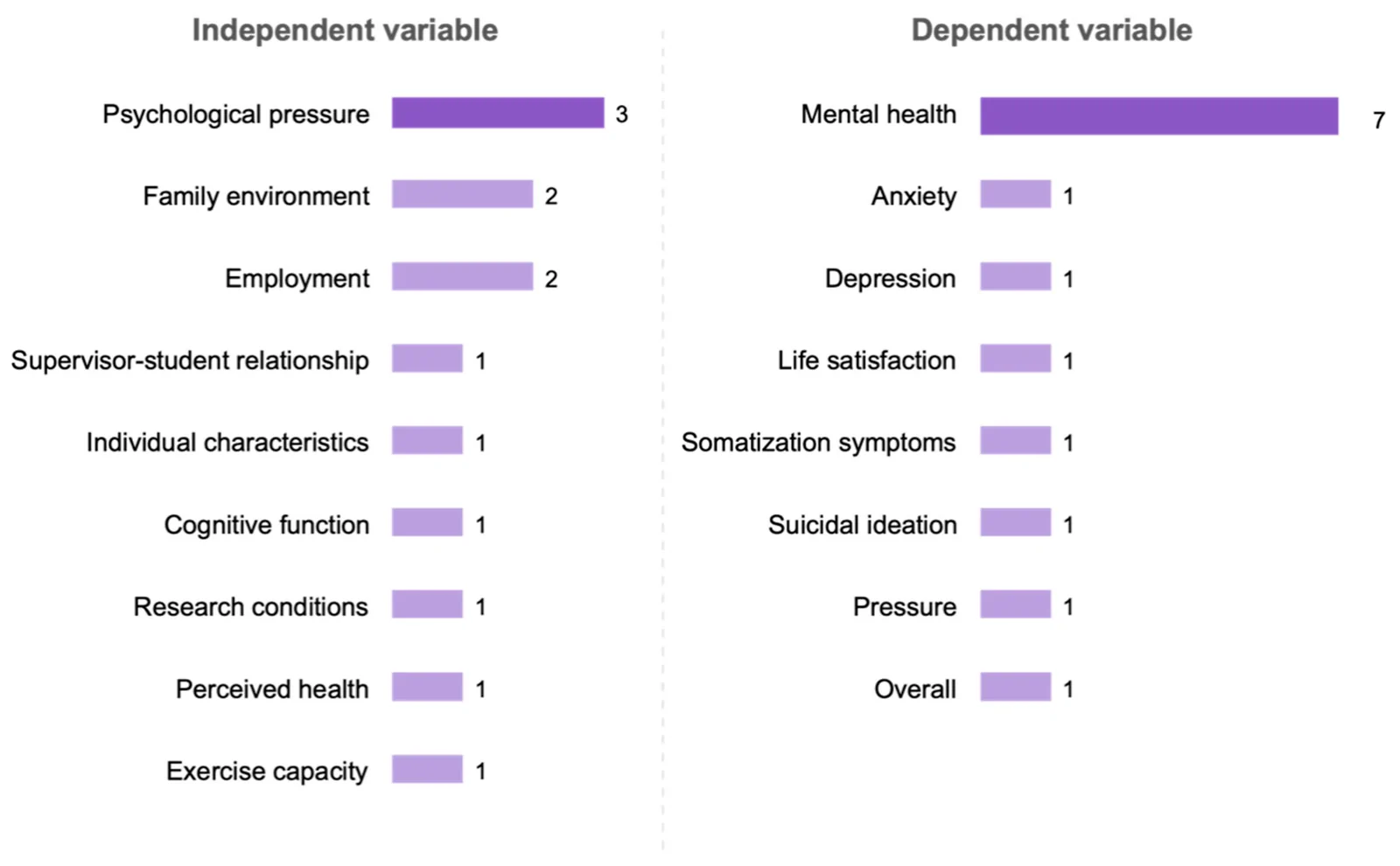
Variables and themes identified in the reviewed studies. Bar length indicates the number of studies. Dark purple highlights the most frequently reported item within each variable category, whereas light purple represents the remaining items.

In the quantitative studies, psychological stress was the most frequently used independent variable (*k* = 3), followed by social support (*k* = 2) and family environmental factors (*k* = 2). Mental health status (*k* = 7) and stress status (*k* = 2) were the most frequent dependent variables. Control variables included individual characteristics such as year of study, gender, and age, whereas mediating variables included resilience-related factors such as optimism and self-efficacy (Figure 3). In the qualitative studies, research topics included anxiety (*k* = 3), stress (*k* = 2), research burnout (*k* = 1), marginal psychology (*k* = 1), depression (*k* = 1), and emotional resilience (*k* = 1).

#### 3.3.4. Data-Analysis Methods and Tools

**Figure 4:**
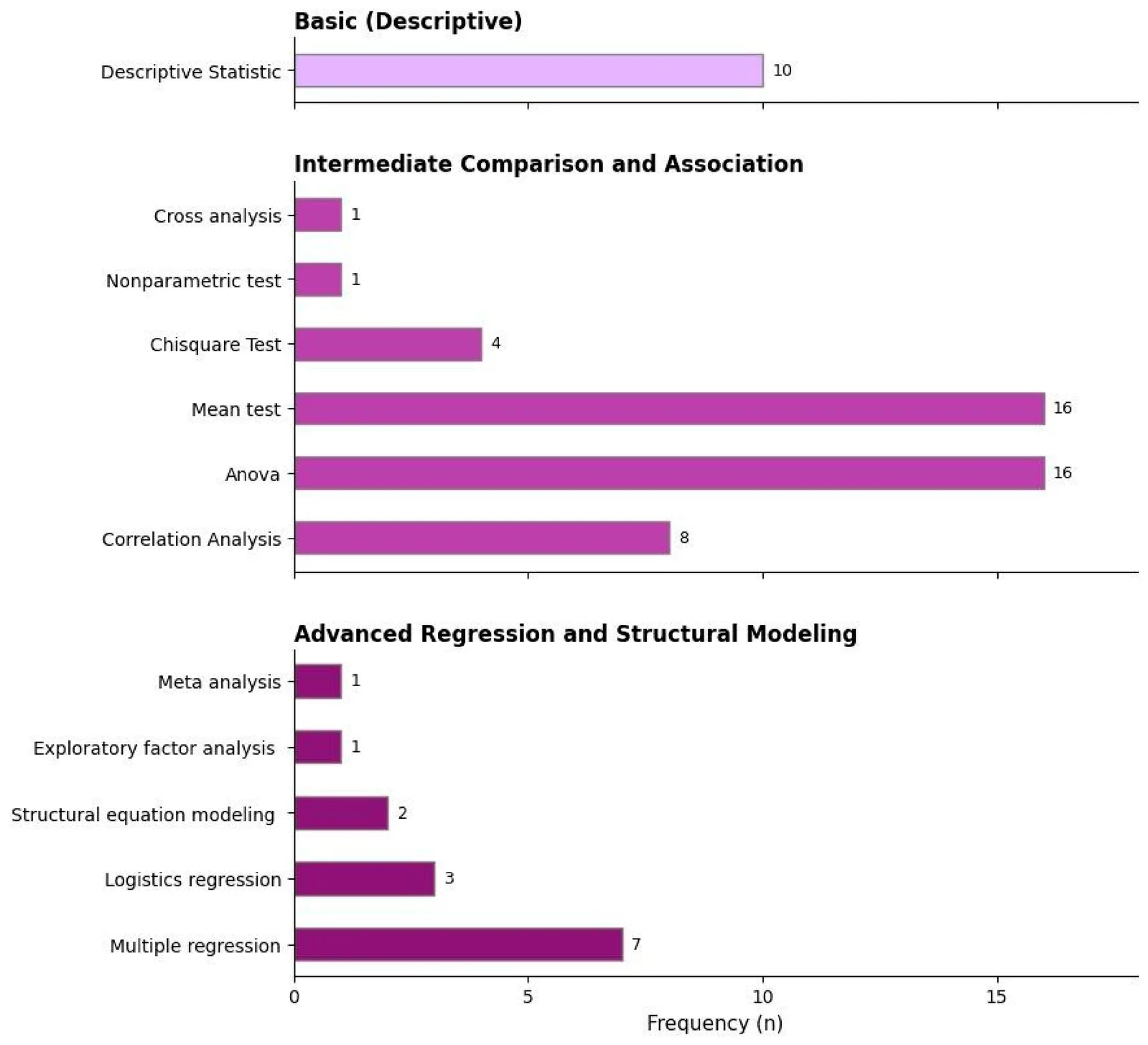
Data analysis methods and tools used in the reviewed studies. Bar length indicates the number of studies. Light, medium, and dark purple represent basic descriptive methods, intermediate comparison and association methods, and advanced regression and structural-modeling methods, respectively.

Mean-comparison tests (*k* = 16) and analysis of variance (ANOVA; *k* = 16) were the most frequently used quantitative analytical methods (Figure 4). Other methods included descriptive statistics (*k* = 10), correlation analysis (*k* = 8), multiple regression analysis (*k* = 7), chi-square tests (*k* = 4), logit regression (*k* = 3), and structural equation modeling (*k* = 2). Three-level coding was the most frequently used qualitative analytical method (*k* = 5).

SPSS was the most frequently used quantitative data-analysis software (*k* = 20), and NVivo 15.3 was the most frequently used qualitative data-analysis software (*k* = 3). Other tools included AMOS 32.0.0.0 (*k* = 2), Visual FoxPro 9.0 Service Pack 2 (SP2) (*k* = 1), EpiData Analysis 3.3.0 (*k* = 1), and Stata 19 (*k* = 1).

#### 3.3.5. Measures and Psychometric Reporting

The review included 28 articles with a quantitative component: 26 quantitative studies and the quantitative portions of two mixed-methods studies. Of these, 26 articles specified their measurement tools, collectively employing 35 measures. Measurement approaches varied: nine studies relied exclusively on established measures, five used self-developed measures, and six used a combination of both. Notably, six studies used scales but did not identify their source or version, highlighting a gap in methodological reporting. Established and self-developed measures are listed in Appendix A Table A2 and Table A3, respectively.

The most frequently used instrument was the Symptom Checklist-90 (SCL-90; Z. Wang, 1984; *k* = 10), followed by the Simplified Coping Style Questionnaire (SCSQ), the Self-Rated Health Measurement Scale (SRHMS), the Social Support Rating Scale (SSRS), and the Family Environment Scale (FES-CV), each used in two studies. Reporting of psychometric properties was inconsistent across the literature. Of the 28 studies that used scales, only nine reported reliability and validity information for all instruments, eight provided this information for some instruments, and the remaining 11 reported no psychometric information.

Additional methodological and reporting characteristics are summarized in Appendix A Table A5. Reporting was uneven: 18 studies did not report their sampling method, four did not report participants’ age or gender, two did not specify their measurement tools, and two did not report their data-analysis methods. These patterns indicate that the broader evidence base is informative for mapping the field but is limited by incomplete reporting of key methodological details.

### 3.4. Participant Characteristics and Content Analysis Findings

#### 3.4.1. Research Sample

**Figure 5:**
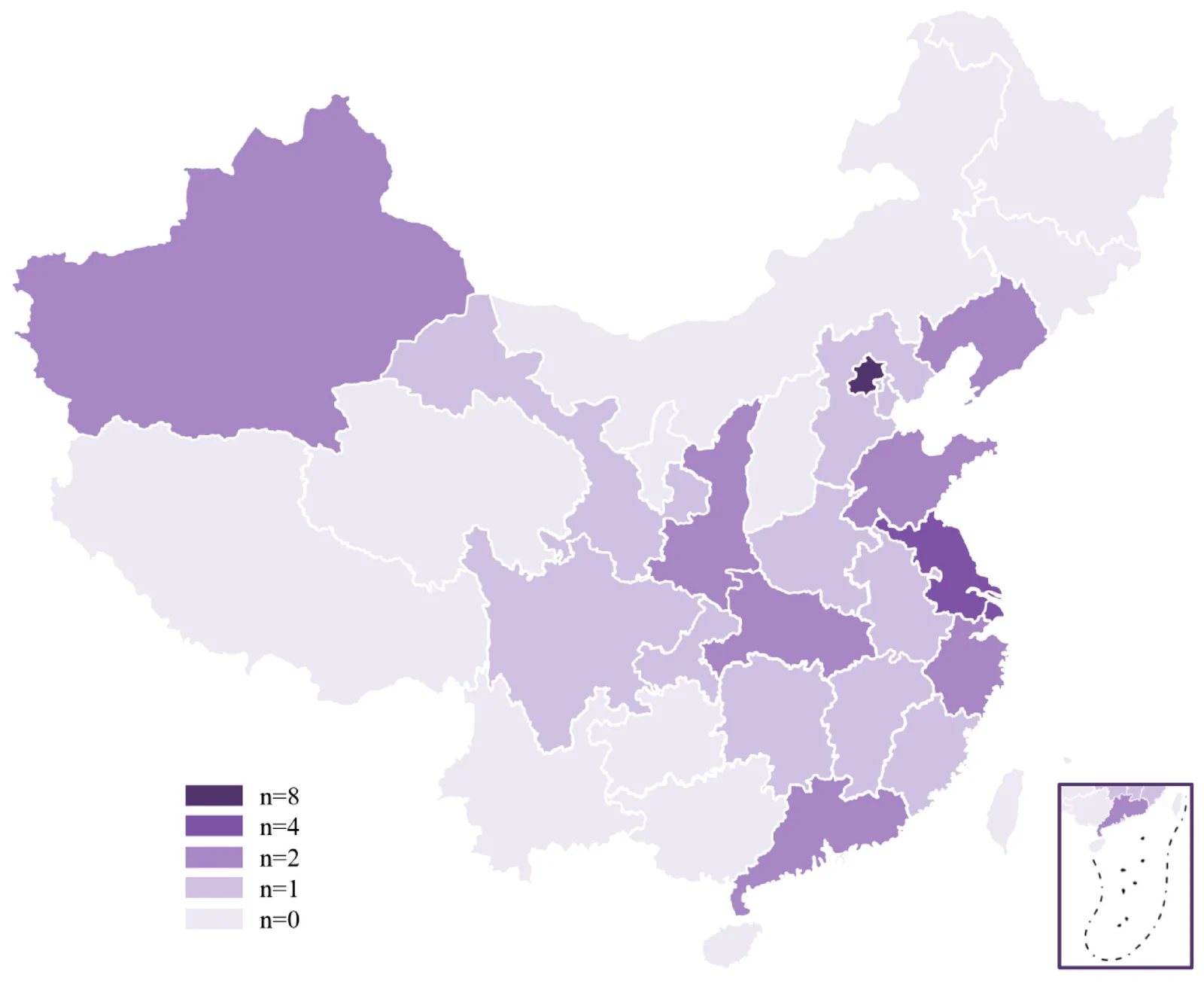
Geographical distribution of participants in the reviewed studies.

Collectively, the reviewed studies included 11,350 doctoral students: 11,101 in quantitative studies and 249 in qualitative studies. To prevent sample duplication, three articles that drew on the same dataset, the Nature Global Doctoral Student Survey, were treated as a single data source. We also mapped the geographical distribution of participants based on the information reported in the studies (Figure 5). Participants were drawn from 20 provinces, municipalities, and autonomous regions. Beijing was represented most frequently (*k* = 8), followed by Shanghai (*k* = 4) and Zhejiang Province (*k* = 4).

The studies used several demographic variables for subgroup comparisons, including gender, age, marital status, year of study, disciplinary background, and training mode. Gender was the most frequent basis for comparison (*k* = 15), followed by year of study and disciplinary background (*k* = 13 each), age (*k* = 11), and marital status and training mode (*k* = 5 each). A detailed breakdown of these frequencies and proportions is provided in Table 3.

#### 3.4.2. Mental Health Findings and Associated Factors

The reviewed studies examined doctoral students’ mental health status, sources of stress, and associated factors. Five studies used the Symptom Checklist-90 (SCL-90) to compare doctoral and master’s students, but their conclusions were inconsistent. One study found that doctoral students had significantly poorer overall mental health than master’s students (X. H. Yang et al., 2007), three found that doctoral students had significantly better mental health (Xing et al., 2005; Hao & Yan, 2014; Gao & Ma, 2016), and one found no significant difference (X. S. Li & Zhu, 2013). In some studies, master’s and doctoral students were analyzed as a combined group, and findings specific to doctoral students appeared only in degree-level subgroup comparisons.

Sources of psychological stress among doctoral students were another focus of the reviewed studies. The main sources were academic and research stress (*k* = 14), employment stress (*k* = 11), financial stress (*k* = 10), stress related to marriage and romantic relationships (*k* = 10), and interpersonal stress (*k* = 9). Additional stressors, each examined in one quantitative study, included the need for achievement, goal attainment, professional interests, sociocultural factors, and environmental change. Qualitative studies also identified age-related pressure, perceived limitations in personal ability, and peer comparison (*k* = 1 each). Factors significantly associated with mental health included social support (*k* = 3), mentoring relationships (*k* = 2), family environment (*k* = 2), personality traits (*k* = 1), coping styles (*k* = 1), and self-efficacy (*k* = 1).

For comprehensive understanding, the summary table for content anaylsis is provided in Table 4.

### 3.5. Risk of Bias in Studies Included in the Meta-Analysis

All five studies met six of the eight applicable JBI criteria and were judged as having some concerns (Appendix A Table A4). The criterion concerning objective measurement of the condition was not met because the studies did not report independent verification of participants’ doctoral-student status using institutional enrollment records or other objective criteria. The principal concern was confounding: although the studies identified or examined relevant demographic characteristics, none reported multi-variable strategies that addressed confounding for the estimates synthesized in this review. In addition, comparisons with Chinese population and youth norms were constructed in this review from aggregate SCL-90 scores, precluding adjustment for individual-level factors such as age, gender, discipline, marital status, and stage of doctoral training. Outcome measurement and the basic statistical analyses were otherwise judged appropriate.

### 3.6. Meta-Analysis Results

**Figure 6:**
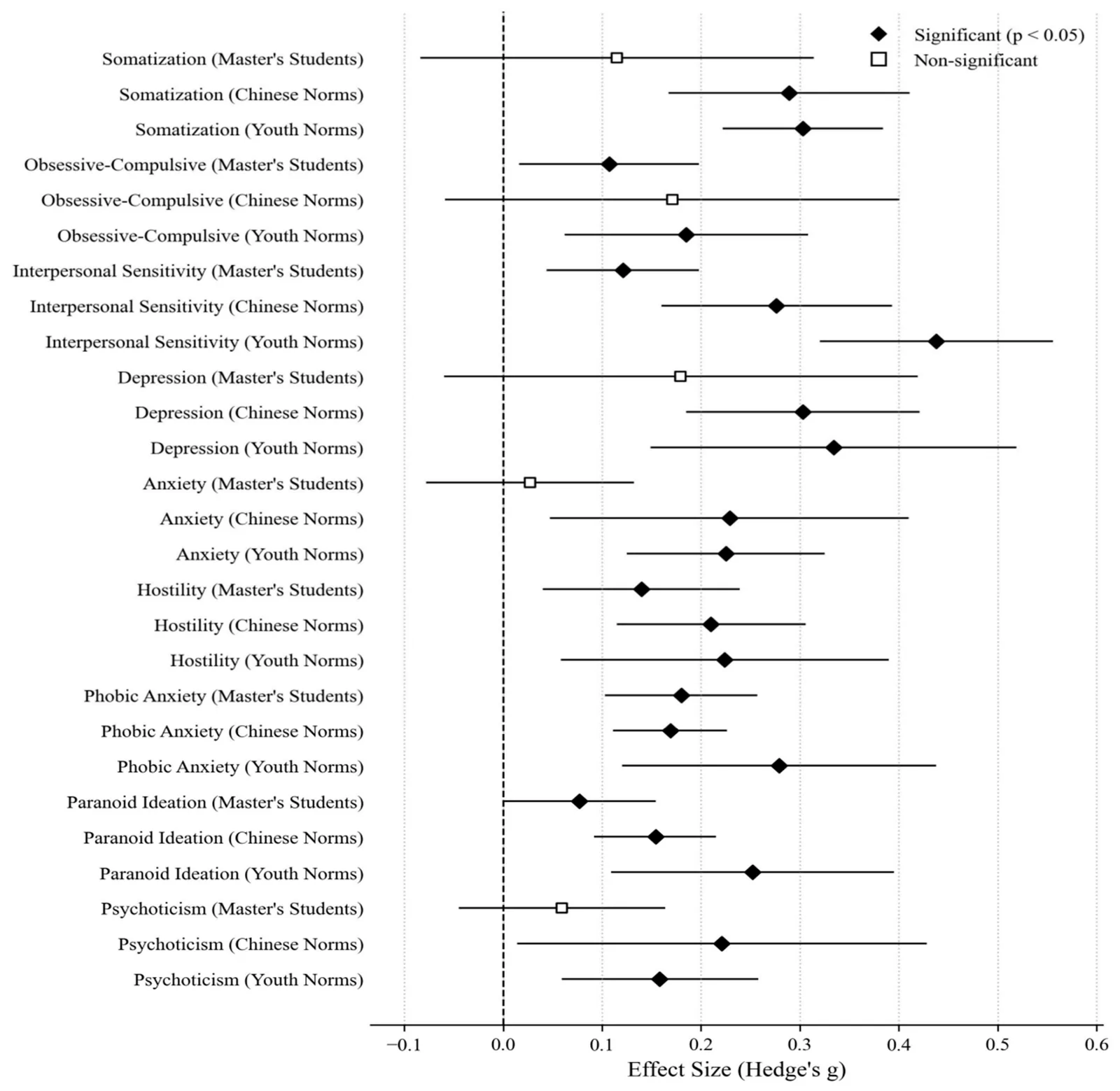
Forest plot of effect sizes for psychological symptoms by comparison group.

To address inconsistent findings concerning the severity of psychological symptoms among Chinese doctoral students, we conducted a meta-analysis of the five studies (*k* = 5) that provided complete SCL-90 data. The studies were published between 2012 and 2017, and their sample sizes ranged from 191 to 1950 participants (Table 1). Based on the content analysis, the meta-analysis examined three comparisons: Chinese doctoral students versus (1) master’s students (*k* = 3 studies), (2) Chinese population norms (*k* = 5), and (3) Chinese youth norms (*k* = 5) (Figure 6). Heterogeneity was assessed using I^2^, τ^2^, and τ. Detailed effect-size estimates across symptom dimensions and reference groups are presented in Table 5, and the corresponding funnel plots are provided in Supplementary SA–SC.

Taken together, the comparative pattern indicates that Chinese doctoral students did not show uniformly poorer mental health than academically similar peers. Differences from master’s students were generally small and confined to several symptom dimensions, whereas elevations were more widespread relative to the Chinese population norms and were evident across all dimensions relative to the youth norms. Interpersonal sensitivity was the only dimension consistently elevated across all three comparisons, while depression and somatization were more prominent in comparisons with the two normative groups. Thus, the apparent extent of doctoral students’ psychological difficulties depended substantially on the reference group, with the clearest disadvantage emerging relative to age-relevant youth norms. The following subsections examine each of these reference-group comparisons in turn.

#### 3.6.1. Comparison with Master’s Students

An analysis of three studies (X. S. Li & Zhu, 2013; Hao & Yan, 2014; Gao & Ma, 2016; total *n* = 5074) revealed small differences between doctoral and master’s students across most psychological symptom dimensions. For most dimensions, the analysis included *k* = 3 studies and *n* = 5074 participants; depression (DEP), psychoticism (PSY), and the total average (T-A) were based on *k* = 2 studies and *n* = 3832 participants. Five of the nine SCL-90 dimensions reached statistical significance, with small positive effect sizes indicating higher symptom scores among doctoral students: phobic anxiety (*g* = 0.180, 95% CI [0.103, 0.257], *p* < 0.001), hostility (*g* = 0.140, *p* = 0.006), interpersonal sensitivity (*g* = 0.121, *p* = 0.002), obsessive-compulsive symptoms (*g* = 0.107, *p* = 0.021), and paranoid ideation (*g* = 0.077, *p* = 0.050). Depression and anxiety, the symptoms most frequently emphasized in the individual studies identified through the content analysis, were not statistically significant in this comparison (*p* = 0.142 and *p* = 0.614, respectively).

Heterogeneity was low for most dimensions (I^2^ = 0% for seven symptoms; τ^2^ = 0 and τ = 0 for these dimensions), although somatization (I^2^ = 84.3%; τ^2^ = 0.026; τ = 0.161) and depression (I^2^ = 89.2%; τ^2^ = 0.040; τ = 0.199) showed substantial between-study variation. However, the low fail-safe N values (0–16) suggest limited robustness.

#### 3.6.2. Comparison with Population Norms

Comparisons with Chinese population norms across all five studies (Lu et al., 2012; X. S. Li & Zhu, 2013; Hao & Yan, 2014; Gao & Ma, 2016; H. M. Guo, 2017) revealed more widespread differences. The analyses included *n* = 2823 participants for most dimensions and *n* = 2320 for anxiety and psychoticism; anxiety, psychoticism, and the total average (T-A) were each based on *k* = 4 studies. Eight of the nine symptom dimensions reached statistical significance, and the positive effect direction indicated that doctoral students had higher symptom scores than the population norms. Depression showed the largest effect (*g* = 0.303, 95% CI [0.185, 0.420], *p* < 0.001), followed by somatization (*g* = 0.289, *p* < 0.001) and interpersonal sensitivity (*g* = 0.276, *p* < 0.001). These findings were consistent with prevalence estimates of mental health problems reported in the content-analysis studies, which ranged from 7.96% to 39.30%. Heterogeneity was high for obsessive-compulsive symptoms (I^2^ = 93.5%; τ^2^ = 0.064; τ = 0.253) and moderate for anxiety (I^2^ = 54.4%; τ^2^ = 0.006; τ = 0.075) and psychoticism (I^2^ = 43.8%; τ^2^ = 0.004; τ = 0.067). Fail-safe N values ranged from 30 to 136.

#### 3.6.3. Comparison with Youth Norms

The most pronounced differences emerged when doctoral students were compared with Chinese youth norms across all five studies (Lu et al., 2012; X. S. Li & Zhu, 2013; Hao & Yan, 2014; Gao & Ma, 2016; H. M. Guo, 2017). All nine symptom dimensions reached statistical significance, and every pooled effect was positive, indicating that doctoral students had higher levels of psychological symptoms than the youth norm group. Interpersonal sensitivity showed a medium effect size (*g* = 0.437, 95% CI [0.319, 0.555], *p* < 0.001), the largest effect observed across all comparisons. Depression (*g* = 0.333, *p* < 0.001) and somatization (*g* = 0.302, *p* < 0.001) also showed higher symptom levels. Heterogeneity was high for depression (I^2^ = 88.4%; τ^2^ = 0.039; τ = 0.198), hostility (I^2^ = 85.6%; τ^2^ = 0.030; τ = 0.174), and phobic anxiety (I^2^ = 84.3%; τ^2^ = 0.027; τ = 0.166), indicating meaningful between-study variability. Fail-safe N values ranged from 15 to 239 across the nine symptom dimensions, with the largest value observed for interpersonal sensitivity.

## 4. Discussion

### 4.1. International Comparison and Unique Contributions

This review contributes to the literature at two complementary levels. First, the systematic review and content analysis synthesize Chinese-language empirical research on doctoral students’ mental health, a body of scholarship that remains largely absent from international reviews relying primarily on English-language sources (Y. Guo & Liu, 2026; Hazell et al., 2020; Jackman et al., 2022). By tracing 35 studies published over nearly two decades, the content analysis documents the development, methodological characteristics, and changing priorities of this research field. It therefore brings culturally situated evidence into the international discussion while providing the basis for the developmental pattern examined further in Section 4.2.

The present findings both converge with and extend previous international reviews. Academic workload, career and financial uncertainty, isolation, belonging, peer support, and supervisory relationships repeatedly appear as correlates of doctoral well-being in international syntheses (Y. Guo & Liu, 2026; Hazell et al., 2020; Jackman et al., 2022; Martínez-García et al., 2024). Our content analysis similarly identified academic, employment, economic, and interpersonal pressures, while our meta-analysis adds quantitative evidence that interpersonal sensitivity was elevated across all reference-group comparisons. This convergence supports viewing doctoral mental health as shaped not only by individual symptoms but also by relational and institutional conditions. Methodologically, the predominance of cross-sectional studies and scarcity of longitudinal and evaluated intervention research in the Chinese literature also mirror limitations identified internationally (Y. Guo & Liu, 2026; Martínez-García et al., 2024).

Second, the meta-analysis contributes symptom-level evidence that the content analysis alone could not provide and helps clarify an inconsistency within the Chinese literature. The content analysis showed that individual studies had reached opposing conclusions about whether doctoral students experienced poorer mental health than master’s students. The pooled findings suggest that differences between doctoral and master’s students were generally small and limited to several symptom dimensions, whereas more dimensions were elevated in comparisons with Chinese population and youth norms. Interpersonal sensitivity was the only symptom dimension consistently elevated across all three reference-group comparisons. This finding should not be interpreted as establishing a symptom unique to Chinese doctoral students; rather, it identifies a relationally salient dimension within the available Chinese SCL-90 evidence. The pooled results also suggest that previously conflicting conclusions may partly reflect small effects, sampling variation, and differences in the symptom dimensions examined. Because only five studies contributed to the meta-analysis, including only three in the doctoral-versus-master’s comparison, these findings should be regarded as preliminary.

The reference-group pattern also offers a developmental interpretation. The relatively small differences between doctoral and master’s students, who share similar academic contexts, suggest that pressures associated with advanced study may be common across graduate education. Larger differences between doctoral students and Chinese youth norms may reflect the combination of prolonged academic dependence and adult life-stage demands (Leick & Köstner, 2025). Consistent with Erikson’s account of early adulthood (Erikson, 1994), doctoral students must negotiate financial, occupational, intimate-relationship, and peer expectations while remaining in uncertain training pathways.

In the Chinese context, these tensions may be intensified by expectations concerning family formation, financial independence, collective achievement, and “face” (mianzi; Dai & Elliot, 2023). This interpretation is plausible rather than causal because the included studies were overwhelmingly cross-sectional and regionally concentrated. Nevertheless, it may help explain why interpersonal sensitivity emerged consistently: concerns about criticism, social comparison, delayed milestones, and relationship strain may be mutually reinforcing. This culturally situated, symptom-dimensional account complements international reviews, which identify supervision and social support as important but generally do not isolate interpersonal sensitivity through comparisons with multiple Chinese reference groups.

### 4.2. Developmental Trajectory of Research on Chinese Doctoral Students’ Mental Health

The content analysis revealed a clear developmental trajectory comprising three distinct stages, which provide important context for interpreting the meta-analytic findings.

Stage 1 (2005–2011): Emergence Phase. During this initial period, doctoral students were rarely studied as a distinct population. Instead, they were typically treated as a subgroup within broader graduate student samples, with findings specific to doctoral students emerging incidentally from demographic subgroup analyses (e.g., Y. C. Xiao et al., 2008). This phase was characterized by descriptive survey research using established scales, with a primary focus on documenting mental health status rather than understanding underlying mechanisms. The tendency to overlook or conflate the characteristics of doctoral students with those of broader graduate student populations is not unique to China and has also been noted in the international literature (Sverdlik et al., 2018). Despite these methodological limitations, studies such as Xing et al. (2005) and X. H. Yang et al. (2007) began to recognize that doctoral students may have a distinct mental health profile that should not be assumed to match those of undergraduates or master’s students.

Stage 2 (2012–2017): Consolidation Phase. This period marked the emergence of doctoral students as a distinct research population. Studies such as Y. Y. Wang et al.’s (2012) investigation of the relationship between personality and stress and You and Chen (2012) work on factors associated with suicidal ideation exemplified this more focused approach. Research objectives expanded from description toward explanation and understanding, supported by increasingly sophisticated statistical techniques, including structural equation modeling. Most studies in the meta-analysis originated from this consolidation phase, reflecting the methodological development of the field.

Stage 3 (2018–2023): Diversification Phase. The most recent period was characterized by substantial growth in publication volume and methodological diversity. Research increasingly focused on potentially vulnerable subgroups, including female doctoral students (He et al., 2018; H. Sun & Zhang, 2020), those in medical specialties (Tan & Chen, 2018), and direct-entry candidates (Hong et al., 2020). This trend aligns with international research recognizing the heightened vulnerability of certain subgroups, particularly female doctoral students (Hazell et al., 2020; Satinsky et al., 2021). The scope of research also expanded to examine constructs such as academic burnout (Y. C. Li, 2020), social anxiety (He et al., 2018), marginal psychology (Hong et al., 2020), and emotional resilience (X. H. Sun & Li, 2023). This phase showed a marked methodological shift toward qualitative and mixed-methods approaches, which increased from 10.5% to 43.8% of publications.

This three-stage evolution helps contextualize the meta-analysis, which drew primarily on Consolidation Phase studies and provided a quantitative synthesis of evidence from the field’s transition toward explanatory research. It also highlights the need for future work that incorporates the more nuanced perspectives emerging during the Diversification Phase.

Several important gaps emerged from the integrated analysis. Geographically, research was concentrated in economically developed eastern regions and major higher-education hubs, including Beijing and Shanghai, limiting understanding of doctoral students’ mental health in less-developed areas where students may face different economic pressures and have access to different social resources. Methodologically, the substantial heterogeneity observed for several symptom dimensions may have arisen from multiple sources. Broad differences in study design or measurement instruments are unlikely to provide the main explanation because all five meta-analytic studies were cross-sectional and used the SCL-90. More plausible sources include variation in sampling and recruitment procedures, sample size, geographical and institutional context, and participant characteristics such as age, gender, discipline, ethnicity, training mode, and stage of doctoral study. In particular, two studies focused on a specific ethnic minority population. For comparisons with population and youth norms, differences in demographic composition and assessment periods between the doctoral samples and normative datasets may also have influenced the estimates. Because only three to five studies contributed to each comparison, formal moderator analyses or meta-regression were not feasible, and these explanations remain tentative. In addition, although mentoring relationships, social support, and institutional environments were frequently identified as important, they were not systematically investigated in quantitative studies. The small number of studies suitable for meta-analysis (*k* = 3–5) further highlights the early stage of rigorous empirical research in this area.

### 4.3. Implications, Limitations, and Future Directions

#### 4.3.1. Implications for University Mental Health Support

The most consistent finding across the meta-analysis was elevated interpersonal sensitivity among doctoral students compared with all reference groups. Together with the content-analysis findings concerning supervisory relationships and social connections, this suggests that interpersonal sensitivity may represent a prominent relational vulnerability associated with doctoral students’ interpersonal difficulties. In doctoral education, it may be reflected in concerns about criticism or rejection, feelings of inadequacy when comparing oneself with peers, discomfort in seeking feedback, and fear of negative evaluation by supervisors or colleagues. These experiences may both affect and be affected by supervisory and peer relationships. Interpersonal sensitivity may also discourage help-seeking because disclosing difficulties can feel exposing or invite anticipated judgment. Universities should therefore address both students’ interpersonal coping and the relational environments in which doctoral training occurs.

Based on these findings, universities could adopt a tiered support approach combining universal prevention for all doctoral students, selective support for students experiencing emerging relational difficulties, and indicated intervention for those experiencing persistent psychological distress. At the universal prevention level, universities should promote relationally supportive doctoral environments. Supervisors should be trained to communicate expectations clearly, provide specific and behavior-focused feedback privately and respectfully, avoid public criticism and unhelpful comparisons, invite questions, and recognize signs of relational distress. Doctoral students should receive training in feedback-seeking, discussing and responding to feedback, assertive communication, boundary setting, conflict resolution, managing social comparison, and help-seeking. Student–supervisor agreements and regular review meetings could clarify expectations and provide structured opportunities to address misunderstandings. Peer mentoring and cohort-based activities could further reduce isolation and excessive competition while strengthening belonging and mutual support.

At the selective-support level, students experiencing emerging supervisory conflict, peer difficulties, or heightened fear of evaluation should have access to mentoring, independently facilitated student–supervisor conversations, interpersonal skills groups, and confidential consultation before these difficulties intensify. At the indicated-intervention level, students experiencing persistent interpersonal distress or associated symptoms such as depression and somatization should have access to confidential counseling and clear referral pathways. Anonymous and online entry points may reduce barriers for students who fear judgment or face-to-face disclosure.

Support should also address the structural pressures identified in the reviewed studies, particularly academic workload, employment uncertainty, financial strain, and tensions between doctoral training and adult life transitions. Graduate schools should establish transparent progression requirements, reasonable workload expectations, emergency financial assistance, career counseling, and flexible leave or extension policies. These measures should be responsive to Chinese doctoral students’ developmental and cultural contexts, including tensions between prolonged academic dependence and expectations concerning career and family progression. Doctoral program staff should be trained to recognize signs of distress and connect students with appropriate support, while qualified mental health professionals should assess whether difficulties require clinical intervention. These initiatives should be evaluated over time to determine whether they improve interpersonal well-being, supervisory and peer relationships, help-seeking, and doctoral progression.

#### 4.3.2. Research Limitations

Several limitations should be considered. First, although the content analysis identified patterns and trends across the literature, it could not explain the causes of contradictory findings. Complementary approaches, such as in-depth qualitative interviews and quantitative causal modeling, are needed to provide deeper explanations.

Second, the language, database, and publication criteria may have introduced selection bias. Although the focus on Chinese-language research was consistent with our aim of introducing underrepresented Chinese scholarship to international discourse, reliance on Chinese databases may have excluded relevant English-language studies. Restricting eligibility to articles published in the Core Journals of China or journals indexed in the Chinese Social Sciences Citation Index also excluded non-indexed journals, dissertations, conference proceedings, and unpublished research. Consequently, the reviewed evidence may disproportionately represent research from established journals and institutions, while studies reporting null, negative, or inconsistent findings may be underrepresented. These restrictions may have affected the observed publication trends, thematic frequencies, and pooled effect estimates. The findings should therefore be interpreted as characterizing indexed Chinese-language scholarship rather than the complete evidence base concerning Chinese doctoral students.

Third, the meta-analysis included only five studies, with three contributing to the comparison with master’s students, limiting the precision of pooled effects and the assessment of heterogeneity and publication bias. The fail-safe N results therefore cannot rule out publication bias. In addition, all studies used the SCL-90, and the JBI assessment identified concerns regarding the lack of adjustment for potential confounders in the aggregate data. Comparisons with external norms may partly reflect demographic or institutional differences rather than doctoral-student status alone. The findings should therefore be regarded as exploratory and may not generalize to other measures, settings, or doctoral-student populations.

Fourth, the evidence base was dominated by cross-sectional survey studies and contained little longitudinal evidence, limiting causal inference and understanding of changes across the doctoral trajectory. Samples were also concentrated in economically developed eastern regions and major higher-education hubs, such as Beijing, Shanghai, and Zhejiang, which may limit generalizability to doctoral students in less-developed and underrepresented regions.

#### 4.3.3. Future Research Directions

The three-stage evolution of research suggests specific priorities for advancing the field. Future research should prioritize longitudinal designs to examine how interpersonal sensitivity and other symptoms evolve across the doctoral trajectory. The consistent finding that interpersonal sensitivity was elevated across comparison groups warrants deeper investigation of the underlying mechanisms, incorporating insights from the growing qualitative literature.

The methodological diversification observed in recent years, with qualitative approaches increasing from 10.5% to 43.8%, creates opportunities for mixed-methods investigations of the sources of heterogeneity observed in the meta-analysis. Research should also expand geographically to include diverse regional contexts and systematically examine potential moderators such as field of study, advisor relationship quality, and institutional support systems.

## 5. Conclusions

This systematic review and meta-analysis provides the most comprehensive examination to date of Chinese-language research on mental health among Chinese doctoral students. The content analysis identified three phases in the development of this field: an Emergence Phase (2005–2011), in which doctoral students were examined as subgroups within broader graduate student populations; a Consolidation Phase (2012–2017), which established doctoral students as a distinct research focus and increasingly adopted explanatory objectives; and a Diversification Phase (2018–2023), characterized by methodological innovation and attention to potentially vulnerable subpopulations. Across these phases, research predominantly emphasized explanation and description, identifying academic stress, employment concerns, and financial pressures as primary risk factors and social support and mentoring relationships as protective factors.

The meta-analysis provided symptom-level evidence, with interpersonal sensitivity showing the most consistent elevation across all comparison groups, followed by depression and somatization. This finding identifies interpersonal functioning, including challenges in supervisory relationships, as a relationally salient area for prevention and intervention. The convergence of the content-analysis and meta-analytic findings underscores the importance of understanding doctoral students’ mental health within its developmental, cultural, and academic contexts and of moving beyond isolated symptoms toward integrated models that address the interconnected academic, interpersonal, and developmental challenges facing this population.

## Figures and Tables

**Table 1:** Study characteristics and sample sizes for the meta-analysis.

Study	Sample Size		Comparison Group(s)	Measures
	Doctoral Students	Master’s Students		
(Lu et al., 2012)	191	N/A	Chinese population/youth norms	SCL-90
(X. S. Li & Zhu, 2013)	225	1725	Master’s students, Chinese population/youth norms	SCL-90
(Hao & Yan, 2014)	503	739	Master’s students, Chinese population/youth norms	SCL-90
(Gao & Ma, 2016)	166	1716	Master’s students, Chinese population/youth norms	SCL-90
(H. M. Guo, 2017)	350	N/A	Chinese population/youth norms	SCL-90

Note: SCL-90 = Symptom Checklist-90. All studies were conducted with Chinese doctoral student populations and formed the basis of the meta-analysis.

**Table 2:** Examples of research objectives in the reviewed studies.

Examples of Research Questions	Research Objective	Study
What are the characteristics of supervisor-induced ostracism?	Exploratory	(Zhou et al., 2020)
What are the current level and prevalence of anxiety among doctoral students?	Descriptive	(Feng, 2021)
What is the relationship among mental health, academic pressure, and doctoral students’ intention to suspend their studies?	Explanatory	(P. Liu & Yang, 2016)
How does marginal psychology develop among early-stage direct-entry doctoral students during the transition period?	Understanding	(Hong et al., 2020)

**Table 3:** Demographic variables used for subgroup comparisons.

Characteristic	Category	*n*	%
Gender	Male	2004	55.0
	Female	1656	45.0
Marital status	Married	431	31.0
	Single	979	69.0
Grade	First year	956	28.0
	Second year	979	28.0
	Third year	729	21.0
	Fourth year and beyond	808	23.0
Disciplinary background	Medicine	506	17.0
	Natural science and engineering	1131	38.0
	Humanities and social sciences	670	23.0
	Military science	637	22.0
Training mode	Full-time	1874	85.0
	Part-time	319	15.0

Note. *n* is for sample size. In total *n* = 3660. Percentages may not sum to 100% due to rounding.

**Table 4:** Summary of findings from the content analysis (*k* = 35).

Domain	Main Content-Analysis Finding	Supporting Evidence
Research designs and methods	The evidence base was predominantly quantitative and survey-based; qualitative and mixed-methods work remained limited, and explicit theoretical frameworks were uncommon.	Quantitative: *k* = 26; qualitative: *k* = 7; mixed methods: *k* = 2; surveys: *k* = 25; interviews: *k* = 7; theoretical frameworks: *k* = 5.
Samples and geographical coverage	The studies included a large combined sample but were concentrated in major higher-education hubs and economically developed eastern regions.	11,350 doctoral students across 20 provincial-level regions; Beijing: *k* = 8; Shanghai: *k* = 4; Zhejiang: *k* = 4.
Measures and psychometric reporting	The SCL-90 was the most common instrument, but reporting of measure sources, versions, reliability, and validity was inconsistent.	SCL-90: *k* = 10; full psychometric reporting: *k* = 9; partial: *k* = 8; none: *k* = 11; measure source/version not reported: *k* = 6.
Doctoral-versus-master’s comparisons	Findings from individual SCL-90 studies comparing doctoral and master’s students were inconsistent.	Doctoral students reported worse mental health: *k* = 1; better mental health: *k* = 3; no significant difference: *k* = 1.
Sources of psychological stress	Academic and career-related pressures were most prominent, alongside financial and relational pressures.	Academic/research: *k* = 14; employment: *k* = 11; financial: *k* = 10; marital/romantic relationships: *k* = 10; interpersonal stress: *k* = 9.
Factors associated with mental health	Relational and social resources were the most frequently identified correlates, followed by family and individual psychological factors.	Social support: *k* = 3; mentoring relationships: *k* = 2; family environment: *k* = 2; personality traits, coping styles, and self-efficacy: *k* = 1 each.

Note. *k* = number of studies. Counts describe how frequently a design, topic, measure, or finding appeared in the 35-study content analysis and should not be interpreted as pooled prevalence estimates.

**Table 5:** Summary of findings from the meta-analysis of SCL-90 symptom dimensions.

Panel A. Doctoral students vs. master’s students										
Statistic	SOM	O-C	I-S	DEP	ANX	HOS	P-A	P-I	PSY	T-A
*k*	3	3	3	2	3	3	3	3	2	2
*n*	5074	5074	5074	3832	5074	5074	5074	5074	3832	3832
*g* (95% CI)	0.115 (−0.084, 0.314)	0.107 (0.016, 0.198)	0.121 (0.044, 0.198)	0.179 (−0.060, 0.419)	0.027 (−0.078, 0.132)	0.140 (0.040, 0.239)	0.180 (0.103, 0.257)	0.077 (0.000, 0.154)	0.059 (−0.045, 0.164)	0.063 (−0.041, 0.168)
*SE*	0.102	0.046	0.039	0.122	0.063	0.051	0.039	0.039	0.053	0.053
Effect direction	Positive	Positive	Positive	Positive	Positive	Positive	Positive	Positive	Positive	Positive
Heterogeneity										
*I*^2^ (%)	84.3	26.5	0.0	89.2	0.0	38.0	0.0	0.0	0.0	0.0
*p*	0.002	0.257	0.394	<0.001	0.994	0.199	0.493	0.407	0.998	0.970
τ^2^	0.026	0.002	0.000	0.040	0.000	0.003	0.000	0.000	0.000	0.000
τ	0.161	0.042	0.000	0.199	0.000	0.054	0.000	0.000	0.000	0.000
Fail-safe N analysis										
*N*_FS_	5	3	3	16	N/A	7	12	0	N/A	N/A
*Z*	3.186	2.621	2.878	4.910	—	3.475	4.378	1.769	—	—
*p*	0.001	0.009	0.004	<0.001	—	<0.001	<0.001	0.077	—	—
Panel B. Doctoral students vs. Chinese population norms										
Statistic	SOM	O-C	I-S	DEP	ANX	HOS	P-A	P-I	PSY	T-A
*k*	5	5	5	5	4	5	5	5	4	4
*n*	2823	2823	2823	2823	2320	2823	2823	2823	2320	2320
*g* (95% CI)	0.289 (0.167, 0.411)	0.171 (−0.059, 0.400)	0.276 (0.160, 0.392)	0.303 (0.185, 0.420)	0.225 (0.125, 0.325)	0.210 (0.115, 0.305)	0.168 (0.111, 0.226)	0.154 (0.092, 0.215)	0.158 (0.059, 0.258)	0.236 (0.166, 0.306)
*SE*	0.062	0.117	0.059	0.060	0.051	0.049	0.029	0.031	0.051	0.036
Effect direction	Positive	Positive	Positive	Positive	Positive	Positive	Positive	Positive	Positive	Positive
Heterogeneity										
*I*^2^ (%)	76.6	93.5	74.1	74.7	54.4	61.7	0.0	9.9	43.8	0.0
*p*	0.002	<0.001	0.004	0.003	0.087	0.034	0.697	0.350	0.149	0.811
τ^2^	0.015	0.064	0.013	0.013	0.006	0.007	0.000	0.000	0.004	0.000
τ	0.121	0.253	0.113	0.115	0.075	0.085	0.000	0.022	0.067	0.000
Fail-safe N analysis										
*N*_FS_	121	39	107	136	42	61	30	30	36	40
*Z*	9.825	5.783	9.256	10.402	6.639	7.110	5.156	6.475	5.605	6.475
*p*	<0.001	<0.001	<0.001	<0.001	<0.001	<0.001	<0.001	<0.001	<0.001	<0.001
Panel C. Doctoral students vs. Chinese youth norms										
Statistic	SOM	O-C	I-S	DEP	ANX	HOS	P-A	P-I	PSY	T-A
*k*	5	5	5	5	4	5	5	5	4	N/A
*n*	2216	2216	2216	2216	1713	2216	2216	2216	1713	N/A
*g* (95% CI)	0.303 (0.221, 0.384)	0.185 (0.062, 0.307)	0.438 (0.319, 0.556)	0.334 (0.149, 0.519)	0.225 (0.125, 0.325)	0.224 (0.058, 0.389)	0.279 (0.120, 0.438)	0.252 (0.109, 0.395)	0.158 (0.059, 0.258)	N/A
*SE*	0.041	0.063	0.060	0.094	0.051	0.085	0.081	0.073	0.051	N/A
Effect direction	Positive	Positive	Positive	Positive	Positive	Positive	Positive	Positive	Positive	N/A
Heterogeneity										
*I*^2^ (%)	40.2	73.7	71.3	88.4	54.4	85.6	84.3	80.5	43.8	N/A
*p*	0.153	0.004	0.007	<0.001	0.087	<0.001	<0.001	<0.001	0.149	N/A
τ^2^	0.003	0.014	0.013	0.039	0.006	0.030	0.027	0.021	0.004	N/A
τ	0.058	0.119	0.113	0.198	0.075	0.174	0.166	0.145	0.067	N/A
Fail-safe N analysis										
*N*_FS_	112	40	239	148	41	63	98	77	15	N/A
*Z*	—	5.830	13.670	10.410	6.570	7.180	8.870	7.930	4.180	N/A
*p*	<0.001	<0.001	<0.001	<0.001	<0.001	<0.001	<0.001	<0.001	<0.001	N/A

Note. Values in the g (95% CI) rows are pooled Hedges’ g estimates, with 95% confidence intervals in parentheses. Positive g values indicate higher SCL-90 symptom scores among doctoral students than in the corresponding reference group. k = number of studies; n = total number of participants; CI = confidence interval; SE = standard error; I^2^ = percentage of observed variation attributable to between-study heterogeneity; τ^2^ = estimated between-study variance; τ = estimated between-study standard deviation; N_FS_ = fail-safe N; Z = standardized test statistic. The *p* values in the Heterogeneity sections refer to tests of between-study heterogeneity, whereas those in the Fail-safe N sections correspond to the reported Z statistics. SOM = somatization; O-C = obsessive–compulsive symptoms; I-S = interpersonal sensitivity; DEP = depression; ANX = anxiety; HOS = hostility; P-A = phobic anxiety; P-I = paranoid ideation; PSY = psychoticism; T-A = total average; N/A = not available; — = not reported.

**Figure A2:**
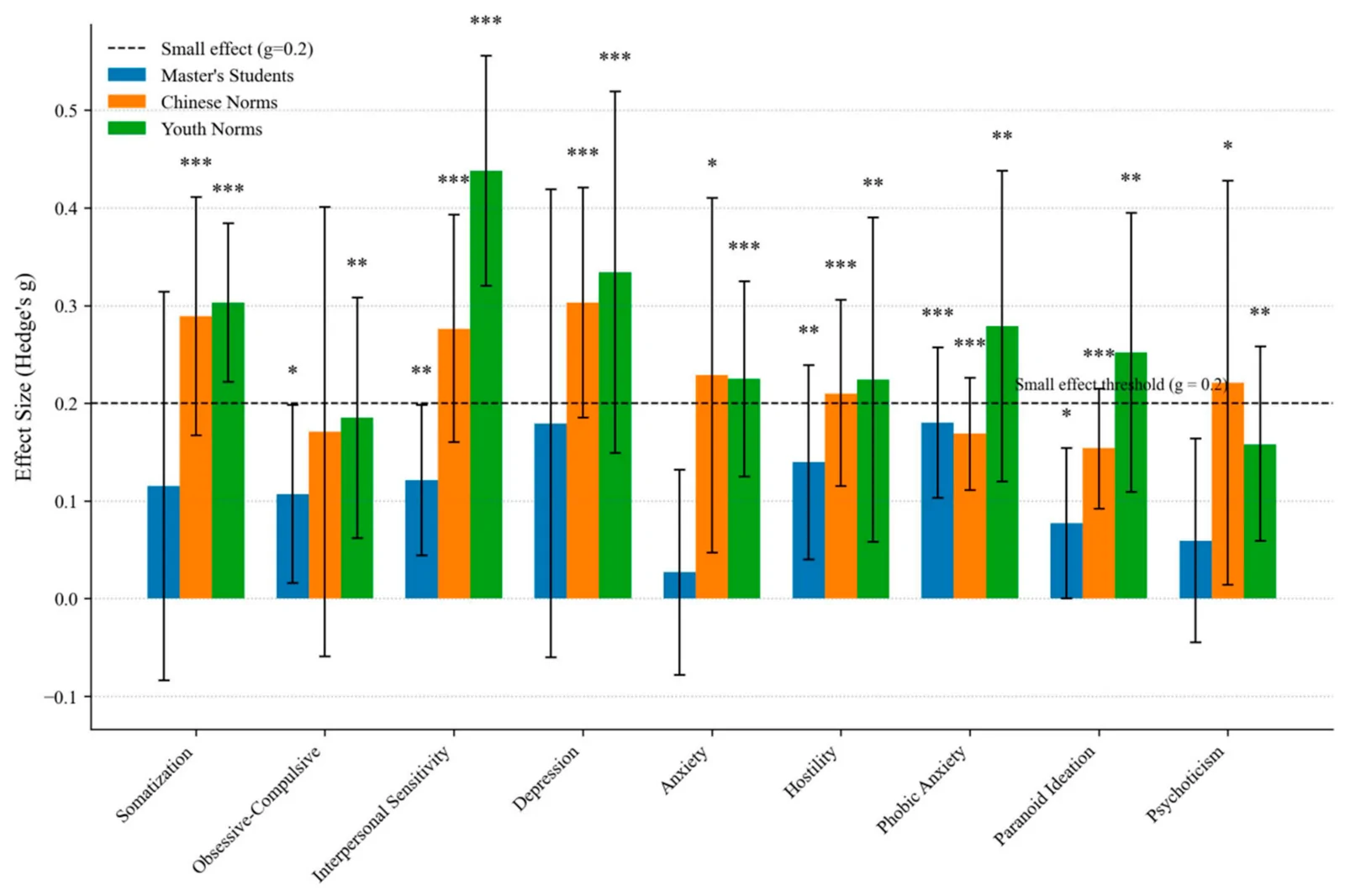
Effect Size Comparison Across Symptom Dimensions and Reference Groups. Bars represent pooled Hedges’ g values, and error bars indicate 95% confidence intervals. Blue, orange, and green bars represent comparisons with master’s students, Chinese population norms, and Chinese youth norms, respectively. Positive values indicate higher symptom scores among doctoral students. The dashed horizontal line indicates the conventional small-effect benchmark (g = 0.20). Asterisks indicate statistical significance based on two-sided tests against zero: * *p* ≤ 0.05, ** *p* < 0.01, and *** *p* < 0.001; no asterisk indicates *p* > 0.05.

## Data Availability

No new primary data were collected for this study. The data analyzed in the systematic review and meta-analysis were extracted from the published studies cited in this article. The content-analysis coding framework is provided in Appendix A. The extracted data supporting the analyses are available from the corresponding author upon reasonable request.

## Supplementary Materials

The following supporting information can be downloaded at: https://www.mdpi.com/article/10.3390/bs16081355/s1, Further explanations of publication bias in the meta-analysis are provided in Supplementary SA–SC.

## Abbreviations

The following abbreviations are used in this manuscript:

CNKI	China National Knowledge Infrastructure
cqVIP	China Science and Technology Journal Database
PRISMA	Preferred Reporting Items for Systematic Reviews and Meta-Analyses
SCL-90	Symptom Checklist-90

## Appendix A

**Table A1:** Content Analysis Framework.

Domains	Codes
Study characteristics	Research objectives, questions, and design
	Target and sample populations
	Data collection methods: sampling methods, measures, reliability and validity
	Data analysis methods
Participant characteristics	Sample size
	Demographic information: gender, age, marital status, year of study, discipline, and training mode
Publication trend	Publication year
	Authors and their occupations
	Grants
Content area	Theoretical models
	Variables: independent, dependent, moderating, mediating, and control variables
	Research findings

**Table A2:** Established Measures Employed in Analyzed Studies.

Measures	Author, Year	Studies (*k*)	Source(s)
Symptom Checklist-90 (SCL-90)	(Z. Wang, 1984)	10	(e.g., X. H. Yang et al., 2007)
Cattell 16 Personality Factor Test (16PF)	(Fan, 2002)	1	(Xing et al., 2005)
Graduate Student Stress Questionnaire	(Yu & Zheng, 2005)	1	(X. H. Yang et al., 2007)
Cognitive Emotion Regulation Questionnaire (CERQ)	(Garnefski et al., 2001)	1	(D. Huang et al., 2008)
Simplified Coping Style Questionnaire (SCSQ)	(Xie, NR)	2	(Y. C. Xiao et al., 2008; Lu et al., 2012)
Physical Activity Rating Scale (PARS-3)	(D. Liang, 1994)	1	(Xia, 2009)
Chinese Big Five Personality Inventory (CBF-PI)	(Hellriegel et al., 2000)	1	(Y. Y. Wang et al., 2012)
General Self-Efficacy Scale (GSES)	(C. Wang et al., 2001)	1	(You & Chen, 2012)
Life Orientation Test-Revised (LOT-R)	(Scheier et al., 1994)	1	(You & Chen, 2012)
Beck Suicide Ideation (BSI)	(X. Li et al., 2010)	1	(You & Chen, 2012)
Social Support Rating Scale (SSRS)	(S. Xiao, 1994)	2	(Lu et al., 2012; H. M. Guo, 2017)
Family Environment Scale (FES)	(Fei et al., 1991)	2	(X. S. Li & Zhu, 2013; Gao & Ma, 2016)
Eysenck Personality Questionnaire (EPQ)	(Y. Gong, 1986)	1	(Y. Wang et al., 2014)
Mental Quality Questionnaire for Army Men (MQQA)	(F. Wang et al., 2007)	1	(Y. Wu et al., 2014)
Perceived Stress Scale (PSS-1)	(Cohen & Williamson, 1988)	1	(P. Liu & Yang, 2016)
Psychological Symptom Scale (PSS-2)	(H. Yang & Liu, 2006)	1	(P. Liu & Yang, 2016)
Self-Rating Depression Scale (SDS)	(Zung, 1965)	1	(J. Sun & Wang, 2021)

Note. *k* = number of included studies that used the measure; NR = publication year not reported in the source study; the original publication could not be located. “Author, Year” refers to the reference cited for the measure, whereas “Source(s)” refers to the included study or studies in which the measure was used.

**Table A3:** Study-Specific Measures Developed in Analyzed Studies.

Measure	Study
Mental Health Factor Questionnaire	(Xing et al., 2005)
Postgraduate Students’ Mental Health Status and Health Education Requirement Questionnaire	(H. Gong, 2011)
Doctoral Students’ Stress Questionnaire	(Y. Y. Wang et al., 2012)
Doctoral Students’ Academic Stress Questionnaire	(You & Chen, 2012)
Suspension Tendency Test	(P. Liu & Yang, 2016)
Self-Assessment Adaptation Questionnaire	(Zang & Zhang, 2016)
Doctoral Students’ Mental Stress Self-Assessment Questionnaire	(H. M. Guo, 2017)
Learning and Living Status Questionnaire	(Tan & Chen, 2018)
Graduate Students’ Comprehensive Health Questionnaire	(Xia et al., 2019)
Anxiety Status and Influencing Factors Questionnaire	(Feng, 2021)
Doctoral Students’ Mental Health Stressors Scale	(J. Sun & Wang, 2021)

**Table A4:** JBI risk-of-bias assessment for the five studies included in the meta-analysis.

Study	Q1	Q2	Q3	Q4	Q5	Q6	Q7	Q8	Overall Judgment
(Lu et al., 2012)	Y	N	Y	Y	Y	N	Y	Y	Some concerns
(X. S. Li & Zhu, 2013)	Y	N	Y	Y	Y	N	Y	Y	Some concerns
(Hao & Yan, 2014)	Y	N	Y	Y	Y	N	Y	Y	Some concerns
(Gao & Ma, 2016)	Y	N	Y	Y	Y	N	Y	Y	Some concerns
(H. M. Guo, 2017)	Y	N	Y	Y	Y	N	Y	Y	Some concerns

Note. Y = Yes; N = No; Q1 = inclusion criteria; Q2 = objective, standard criteria for measurement of the condition; Q3 = valid and reliable exposure measurement; Q4 = valid and reliable outcome measurement; Q5 = confounding factors identified; Q6 = strategies to address confounding; Q7 = appropriate statistical analysis; Q8 = participants and setting described in detail. Q2 was rated N because the studies did not recruit participants on the basis of a clinical diagnosis. Overall judgments were based on the pattern and importance of item-level judgments rather than a numerical cutoff. The primary concern was the absence of strategies to address confounding in the aggregate comparisons synthesized in the review.

**Table A5:** Methodological and reporting characteristics of studies included in the content analysis (*k* = 35).

#	Study	Research Design	Target Population	Sample Size (*N*); Doctoral Students (*n*)	Age; Gender	Sampling Method	Measures	Data-Analysis Methods
1	(Xing et al., 2005)	Quan	Postgraduate medical students	*N* = 728; *n* = 187	Mean age = 31.02 ± 7.09; female = 31.87%	Not reported	SCL-90, 16PF, SQ	1, 2, 3
2	(Duan, 2007)	Qual	Doctoral students	*N* = *n* = 10	Female = 50%	Not reported	Not reported	Not reported
3	(X. H. Yang et al., 2007)	Quan	Postgraduate students	*N* = 2681; *n* = 339	Not reported	Cluster sampling	SCL-90, GSSQ	2, 3, 4, 5
4	(D. Huang et al., 2008)	Quan	Postgraduate students	*N* = 532; *n* = 67	Female = 57.04%	Not reported	CERQ	3
5	(Y. C. Xiao et al., 2008)	Quan	Postgraduate students	*N* = 1061; *n* = Not reported	Female = 44.5%	Not reported	SCSQ	1, 2, 3
6	(Xia, 2009)	Quan	Postgraduate students	*N* = 1256; *n* = 245	Mean age = 25.71; Female = 43.9%	Cluster sampling	PARS-3, SRHMS	2, 3
7	(H. Gong, 2011)	Quan	Postgraduate students	*N* = 551; *n* = 156	Female = 43.9%	Not reported	SQ	1
8	(Y. Y. Wang et al., 2012)	Quan	Doctoral students	*N* = *n* = 119	Mean age = 29.14 ± 3.56; female = 60.50%	Random sampling	SQ, CBF-PI	1, 2, 3, 6
9	(You & Chen, 2012)	Quan	Doctoral students	*N* = *n* = 375	Female = 57.1%	Random sampling	GSES, LOT-R, BSI, SQ	1, 2, 3, 6
10	(Lu et al., 2012)	Quan	Doctoral students	*N* = *n* = 191	Female = 50.26%	Random sampling	SCL-90. SCSQ, SSRS	1, 2, 3, 5, 6
11	(X. S. Li & Zhu, 2013)	Quan	Postgraduate students	*N* = 1950; *n* = 225	Mean age = 25.1; female = 42.26%	Not reported	SCL-90, FES-CV	2, 5, 6
12	(Hao & Yan, 2014)	Quan	Postgraduate students of a university in Xi’an	*N* = 1245; *n* = 503	Female = 41.9%	Stratified sampling	SCL-90	2
13	(Y. Wang et al., 2014)	Quan	Postgraduate military students	*N* = 1536; *n* = 464	Mean age = 26.65 ± 3.53; female = 38.1%	Cluster sampling	SCL-90, EPQ	2, 3, 4
14	(Y. Wu et al., 2014)	Quan	Postgraduate military students	*N* = 749; *n* = 173	Mean age = 27.01 ± 4.01; female = 37.7%	Cluster sampling	SCL-90, MQQA	2, 3
15	(Gao & Ma, 2016)	Quan	Uyghur university students	*N* = 1882; *n* = 166	Mean age = 25.23 ± 5.63; female = 40.28%	Cluster sampling	SCL-90, FES-CV	2, 4, 5, 6
16	(P. Liu & Yang, 2016)	Quan	Postgraduate students	*N* = 591; *n* = 148	Mean age = 23.7 ± 2.2; female = 66.84%	Not reported	PSS-1, PSS-2, SQ	5, 6
17	(Zang & Zhang, 2016)	Quan	Postgraduate students	*N* = 7981; *n* = 1302	Female = 36.60%	Not reported	SCL-90, SQ	2, 3
18	(Feng & Zhang, 2017)	Qual	Doctoral students	*N* = *n* = 28	Female = 46.43%	Convenience sampling	Semi-structured interview	7
19	(H. M. Guo, 2017)	Quan	Doctoral students	*N* = *n* = 350	Female = 40%	Not reported	SCL-90, SSRS, SQ	2, 3, 5, 6
20	(He et al., 2018)	MMR	Female doctoral students	*N* = *n* = 50(6)	Female = 100%	Convenience sampling	Non-structured interview; Not reported	Not reported
21	(Tan & Chen, 2018)	Quan	Postgraduate medical students	*N* = *n* = 315	Female = 47.31%	Cluster sampling	SQ, SAS	2, 3, 4, 8
22	(Xia et al., 2019)	Quan	Postgraduate students	*N* = 7385; *n* = 1646	Female = 50.9%	Not reported	SRHMS, SQ	2, 3, 9
23	(Song et al., 2019)	Quan	Postgraduate students	*N* = 5747; *n* = 1708	Not reported	Random sampling	N/A	1, 5, 10
24	(Y. C. Li, 2020)	Qual	Doctoral students	*N* = *n* = 107	Female = 43.93%	Not reported	Semi-structured interview	7
25	(Zhou et al., 2020)	Quan	Postgraduate students	*N* = 864; *n* = 131	Mean age = 24.37 ± 2.02; female = 53.24%	Not reported	SIOQ, LSS, CES-D-10, Buss-Perry	2, 3, 11
26	(H. Sun & Zhang, 2020)	Qual	Female doctoral students	*N* = *n* = 13	Mean age = 32.31; female = 100%	Snowball sampling	Semi-structured interview	7
27	(Hong et al., 2020)	Qual	Female doctoral students	*N* = *n* = 22	Female = 50%	Not reported	Semi-structured interview	7
28	(Feng, 2021)	MMR	Doctoral students	*N* = *n* = 1140	Female = 35.61%	Cluster sampling	SAS, SQ, semi-structured interview	7, 12
29	(J. Sun & Wang, 2021)	Quan	Doctoral students	*N* = *n* = 938	Female = 44.88%	Not reported	SDS, SQ	1, 3
30	(Jin & Yang, 2021)	Quan	Doctoral students	*N* = *n* = 6320	Not reported	Not reported	N/A	10, 13, 14
31	(Xie & Cai, 2022)	Quan	Doctoral students	*N* = *n* = 698	Not reported	Not reported	N/A	13
32	(J. Zhang & Zhao, 2022)	Quan	Doctoral students	*N* = *n* = 620	Female = 50.6%	Not reported	N/A	1, 13
33	(Cheng & Li, 2022)	Qual	Doctoral students experienced depression	*N* = *n* = 14	Female = 64.29%	Convenience sampling	In-depth interview	15
34	(Deng & Wang, 2023)	Quan	Postgraduate students	*N* = 1034; *n* = 191	Mean age = 24.6; female = 56.7%	Not reported	SQ	1, 6, 11
35	(X. H. Sun & Li, 2023)	Qual	Doctoral students	*N* = *n* = 21	Female = 47.62%	Snowball sampling	Semi-structured and informal interview	16

Note. *N* = total sample size; *n* = number of doctoral students; Quan = quantitative research; Qual = qualitative research; MMR = mixed-methods research; N/A = not applicable. Measure abbreviations: SCL-90 = Symptom Checklist-90; 16PF = Cattell 16 Personality Factor Questionnaire; GSSQ = Graduate Student Stress Questionnaire; CERQ = Cognitive Emotion Regulation Questionnaire; SCSQ = Simplified Coping Style Questionnaire; PARS-3 = Physical Activity Rating Scale; SRHMS = Self-Rated Health Measurement Scale; CBF-PI = Chinese Big Five Personality Inventory; GSES = General Self-Efficacy Scale; LOT-R = Life Orientation Test-Revised; BSI = Beck Scale for Suicide Ideation; SSRS = Social Support Rating Scale; FES-CV = Family Environment Scale-Chinese Version; EPQ = Eysenck Personality Questionnaire; MQQA = Mental Quality Questionnaire for Army Men; PSS-1 = Perceived Stress Scale; PSS-2 = Psychological Symptom Scale; SIOQ = Supervisor-Induced Ostracism Questionnaire; LSS = Life Satisfaction Scale; CES-D-10 = 10-item Center for Epidemiologic Studies Depression Scale; SAS = Self-Rating Anxiety Scale; SDS = Self-Rating Depression Scale. Data-analysis codes: 1 = descriptive statistics; 2 = mean test; 3 = ANOVA; 4 = chi-square test; 5 = multiple regression analysis; 6 = correlation analysis; 7 = grounded theory; 8 = nonparametric test; 9 = meta-analysis; 10 = cross-tabulation; 11 = structural equation modeling; 12 = exploratory factor analysis; 13 = logistic regression; 14 = stepwise regression; 15 = in-depth interview; 16 = thematic analysis.
