# Supplementary material for: More than Depression: Doctoral Students’ Mental Health in China: A Systematic Review and Meta-Analysis
Source: Behav Sci (Basel). 2026 Aug 6;16(8):1355. doi: 10.3390/bs16081355 (PMC13509584; doi:10.3390/bs16081355)
Supplement: Supplementary file 1 [file behavsci-16-01355-s001.zip › behavsci-4428905-supplementary.pdf]

Supplementary SA Funnel plots for Doctoral Students & Master Students Group

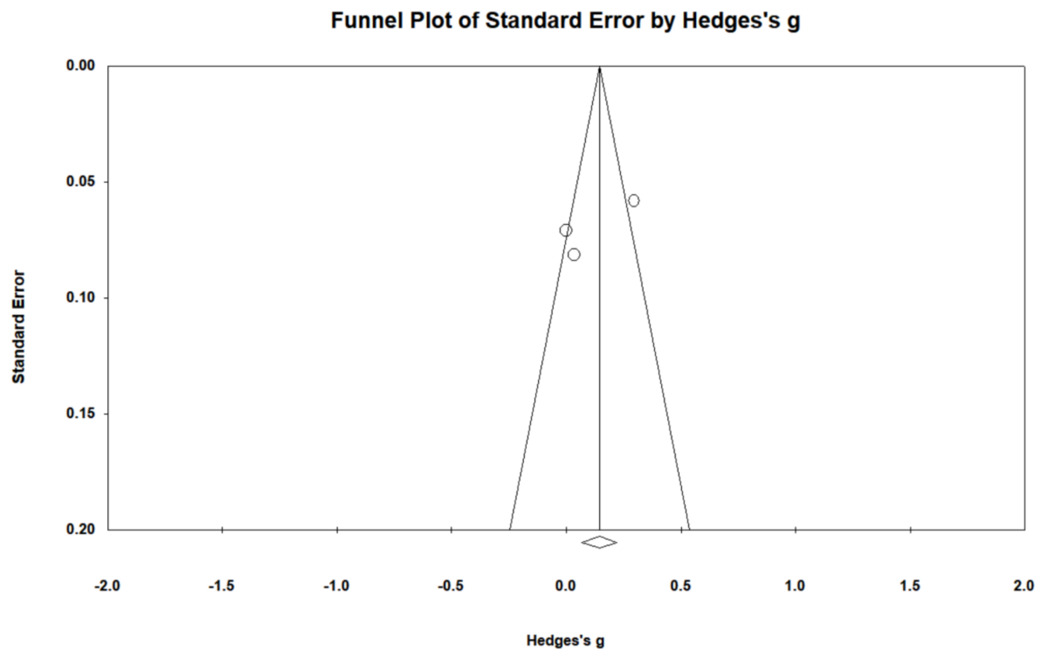

Figure SA1. DS & MS Group SOM funnel plot

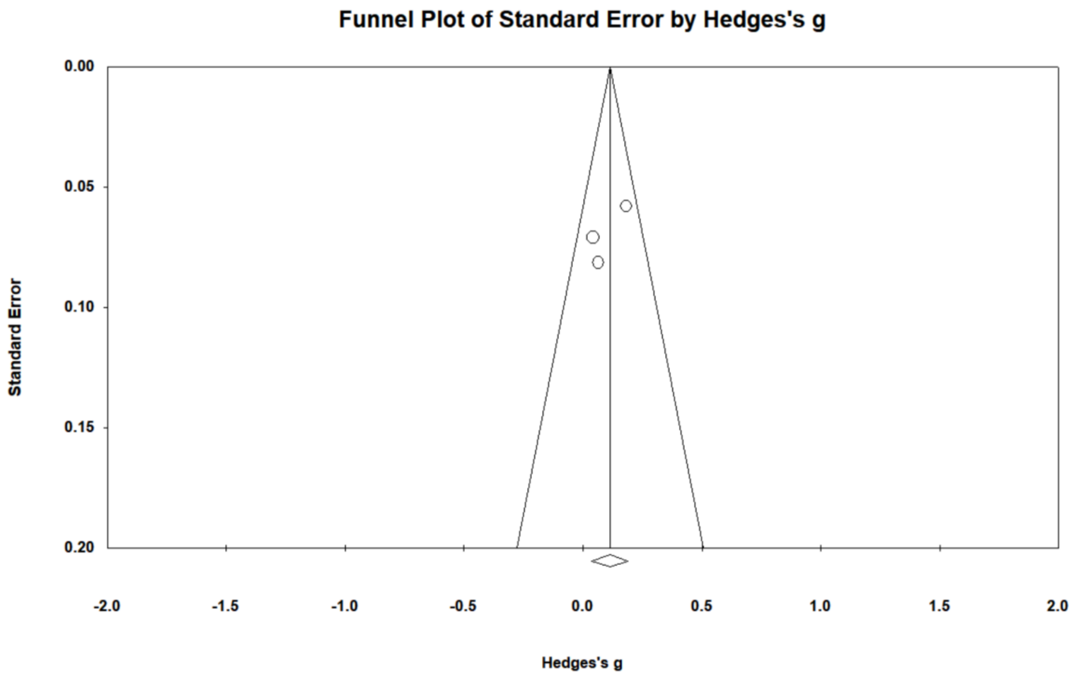

Figure SA2. DS & MS Group O-C funnel plot

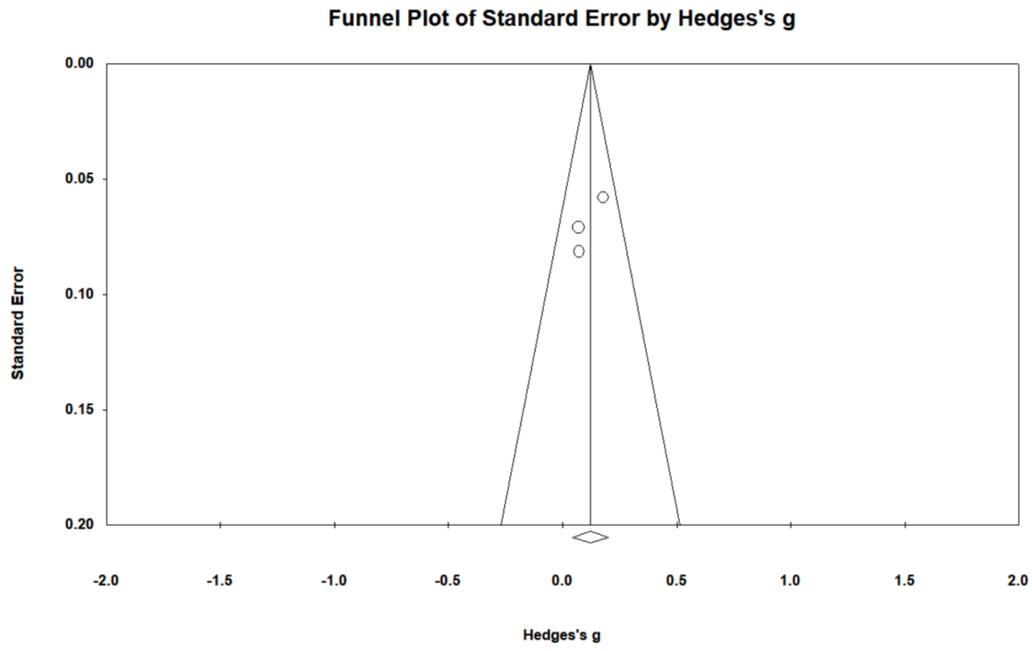

Figure SA3. DS & MS Group I-S funnel plot

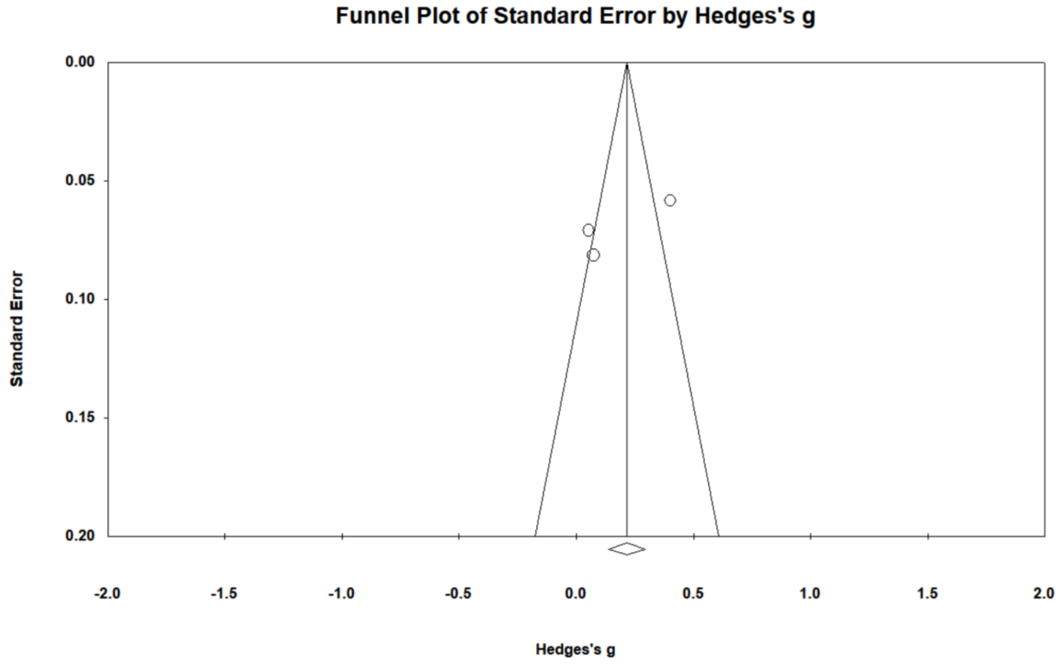

Figure SA4. DS & MS Group DEP funnel plot

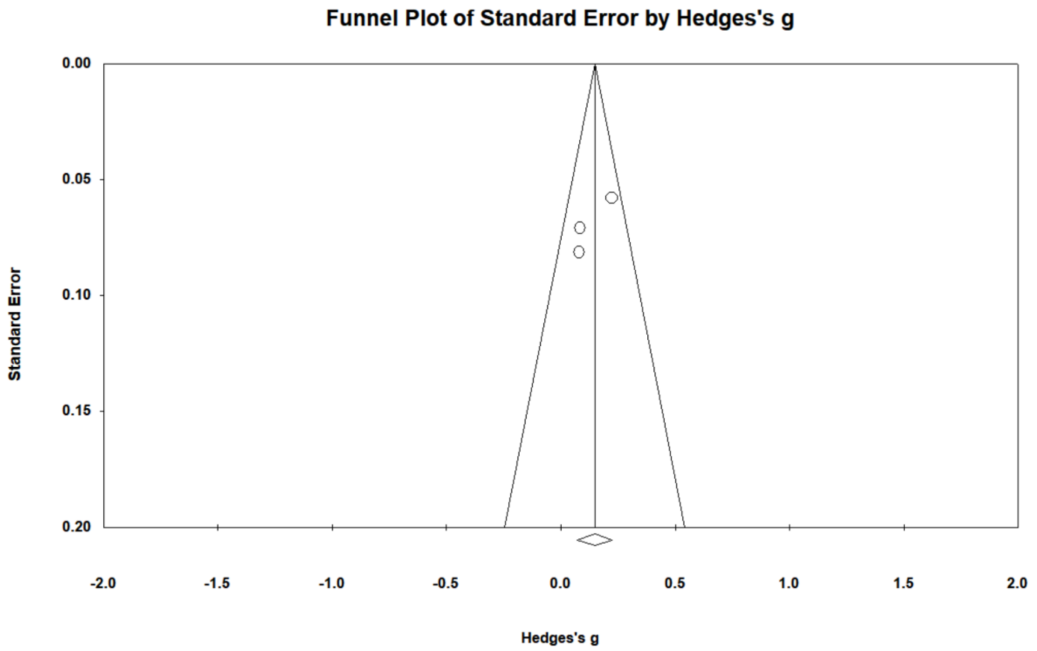

Figure SA5. DS & MS Group HOS funnel plot

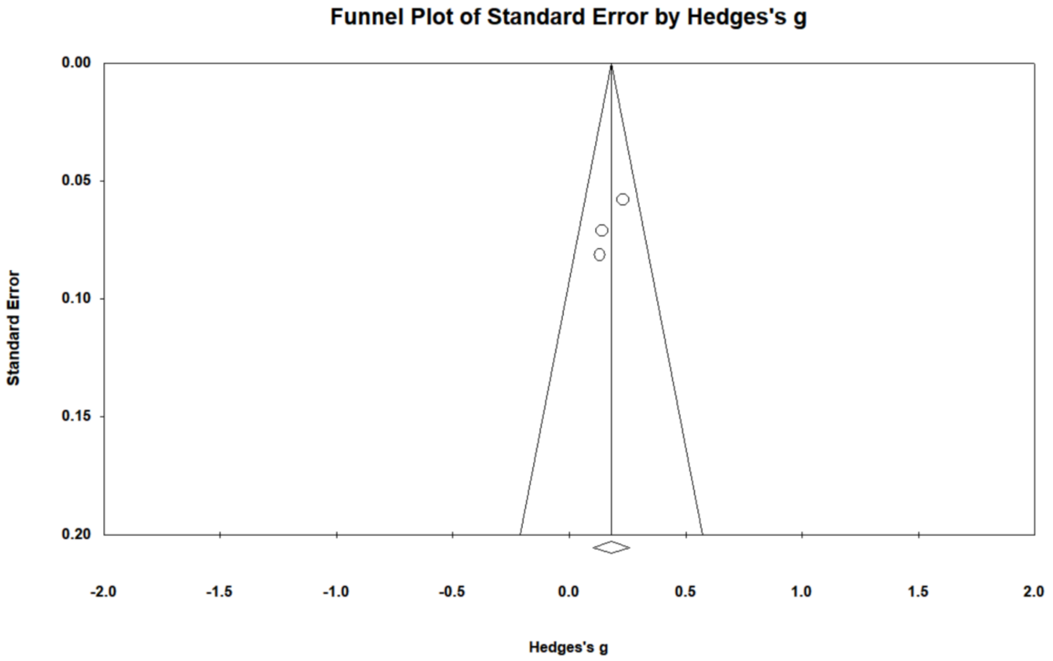

Figure SA6. DS & MS Group P-A funnel plot

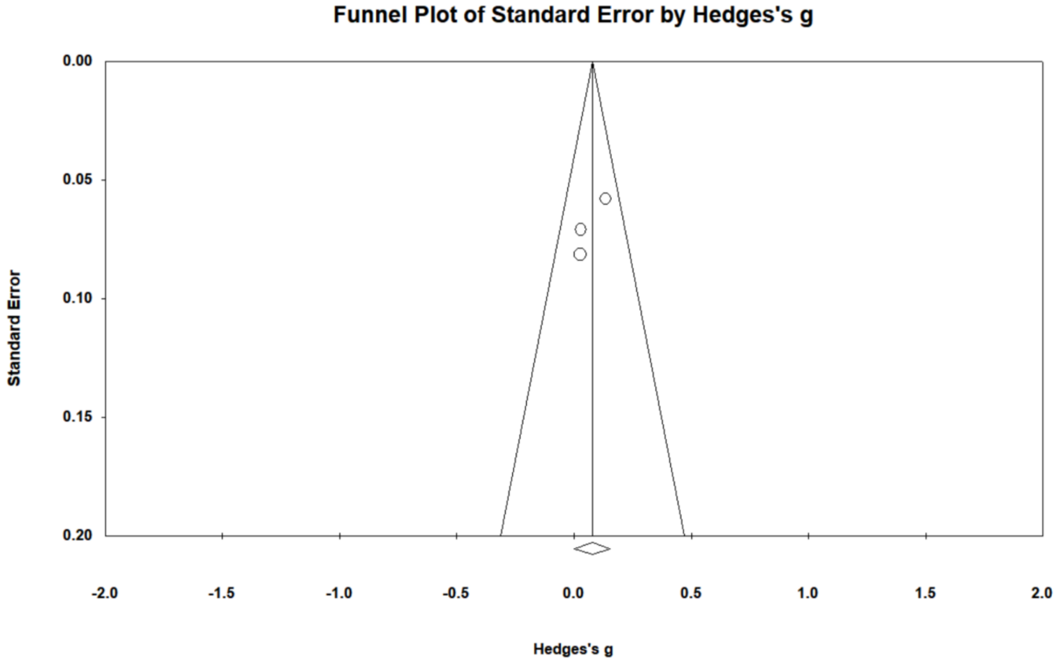

Figure SA7. DS & MS Group P-I funnel plot

Supplementary SB Funnel plots for Doctoral Students & Chinese Norms Group

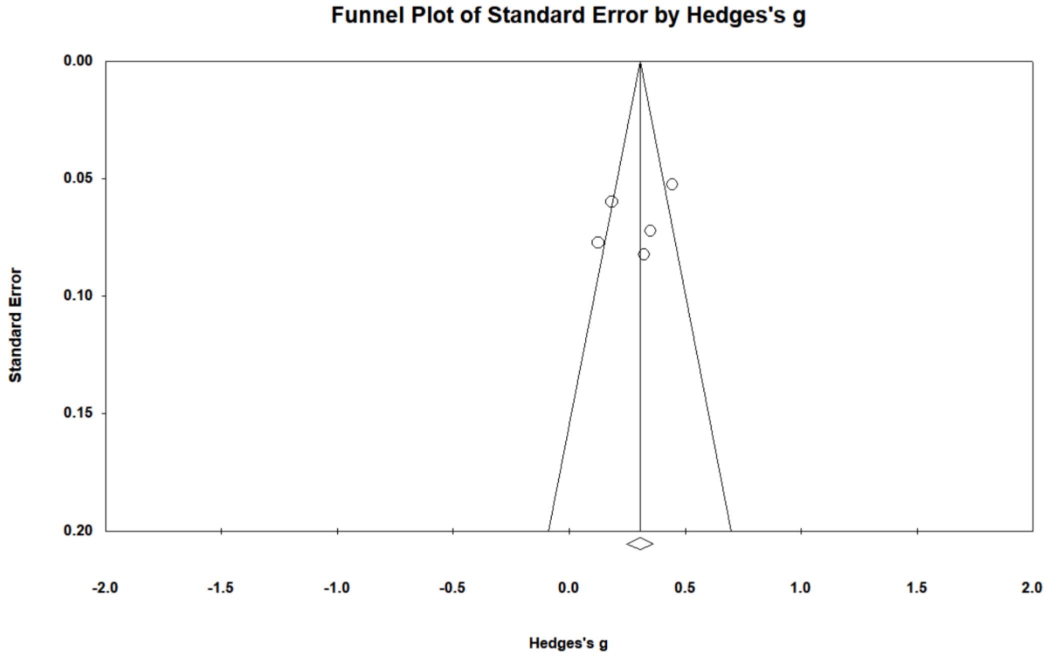

Figure SB1. DS & CN Group SOM funnel plot

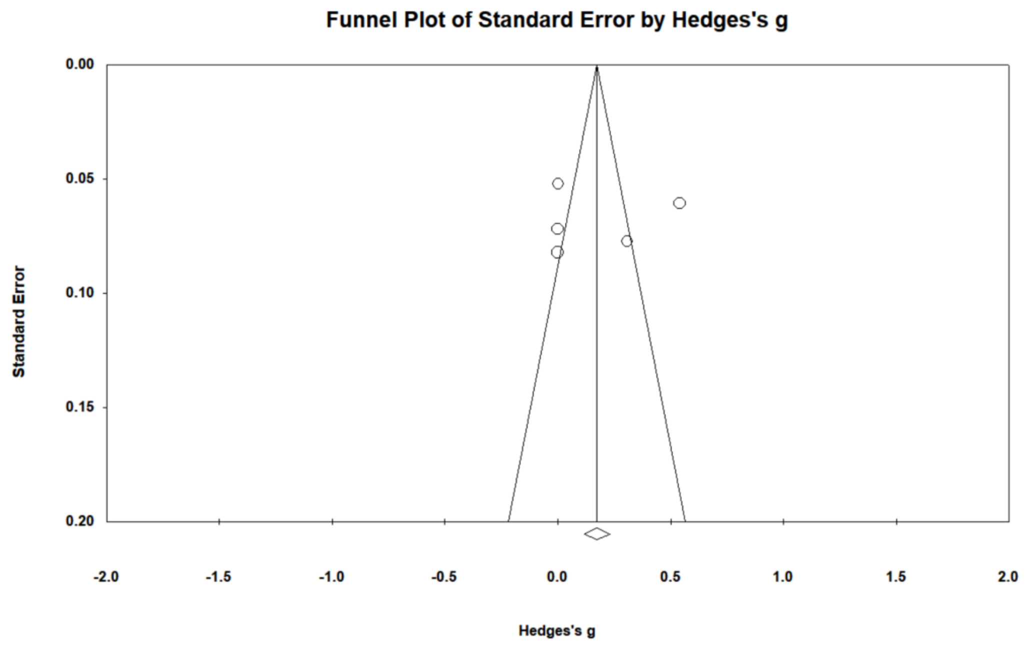

Figure SB2. DS & CN Group O-C funnel plot

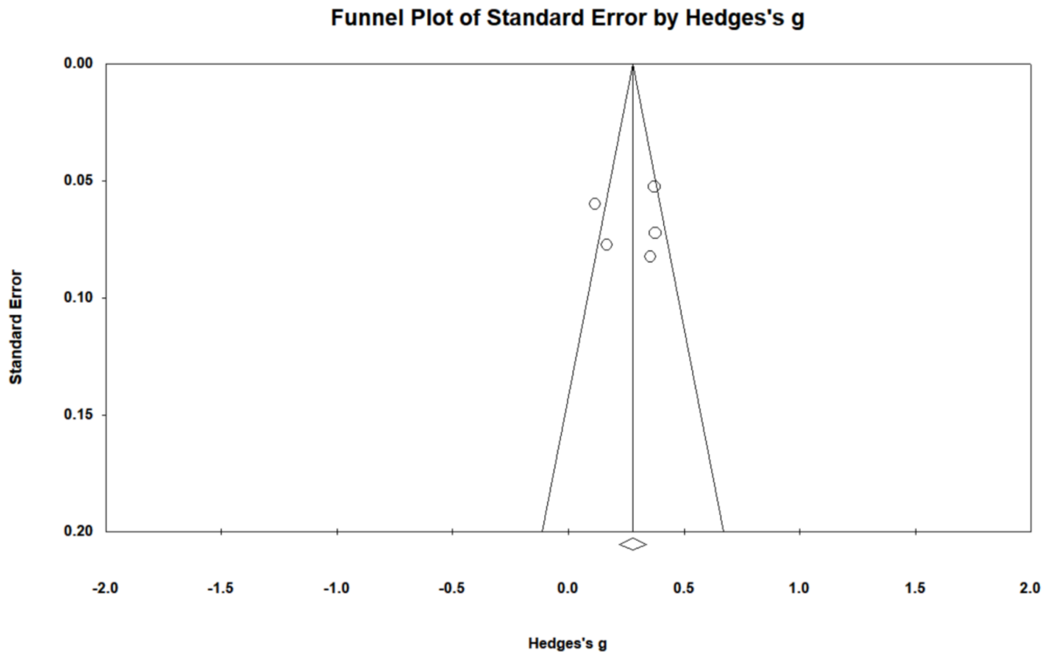

Figure SB3. DS & CN Group I-S funnel plot

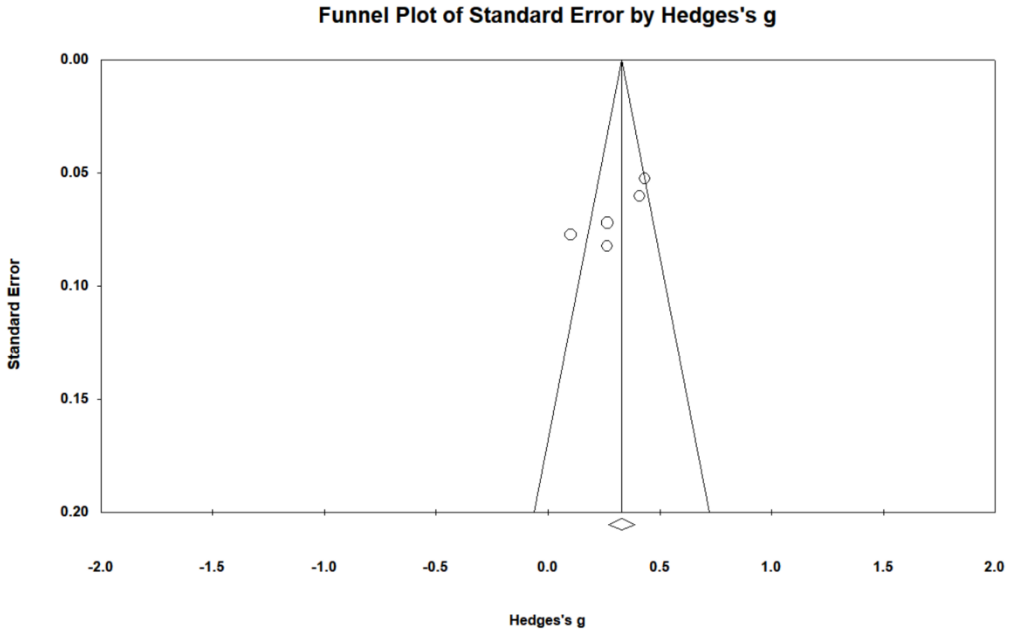

Figure SB4. DS & CN Group DEP funnel plot

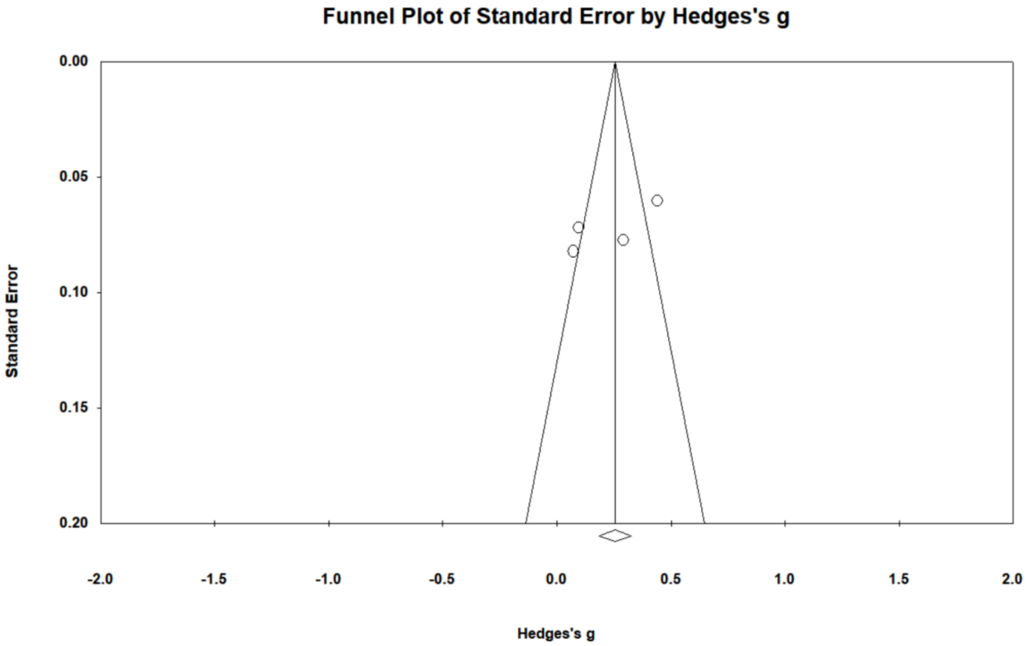

Figure SB5. DS & CN Group ANX funnel plot

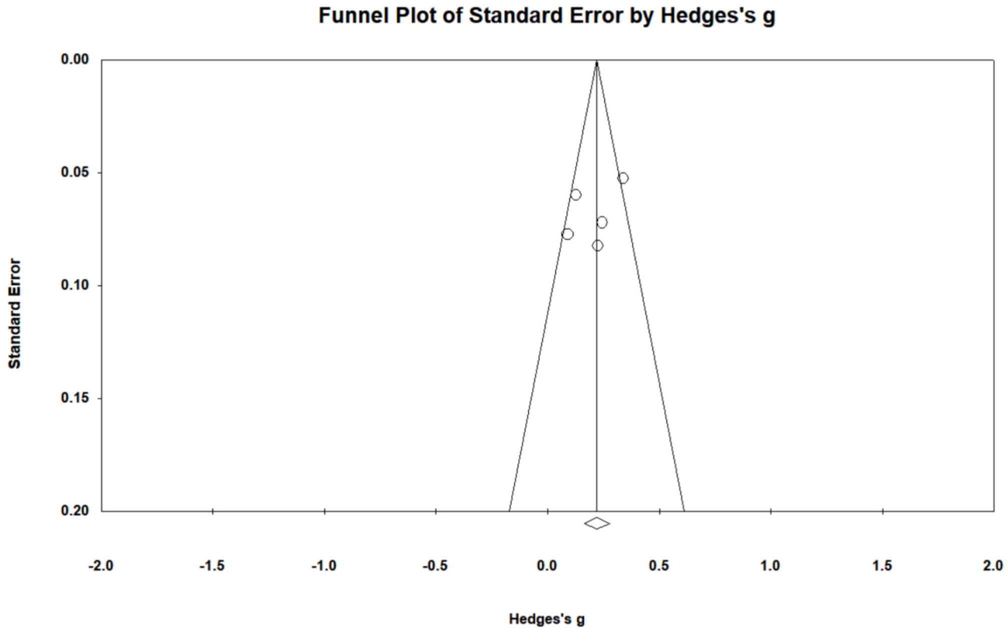

Figure SB6. DS & CN Group HOS funnel plot

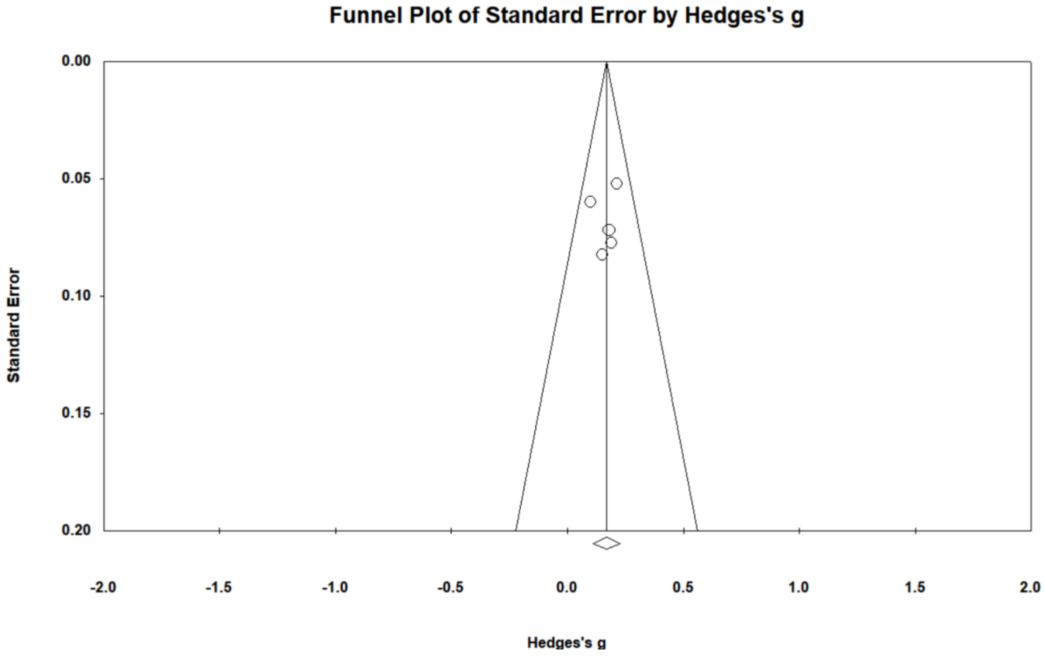

Figure SB7. DS & CN Group P-A funnel plot

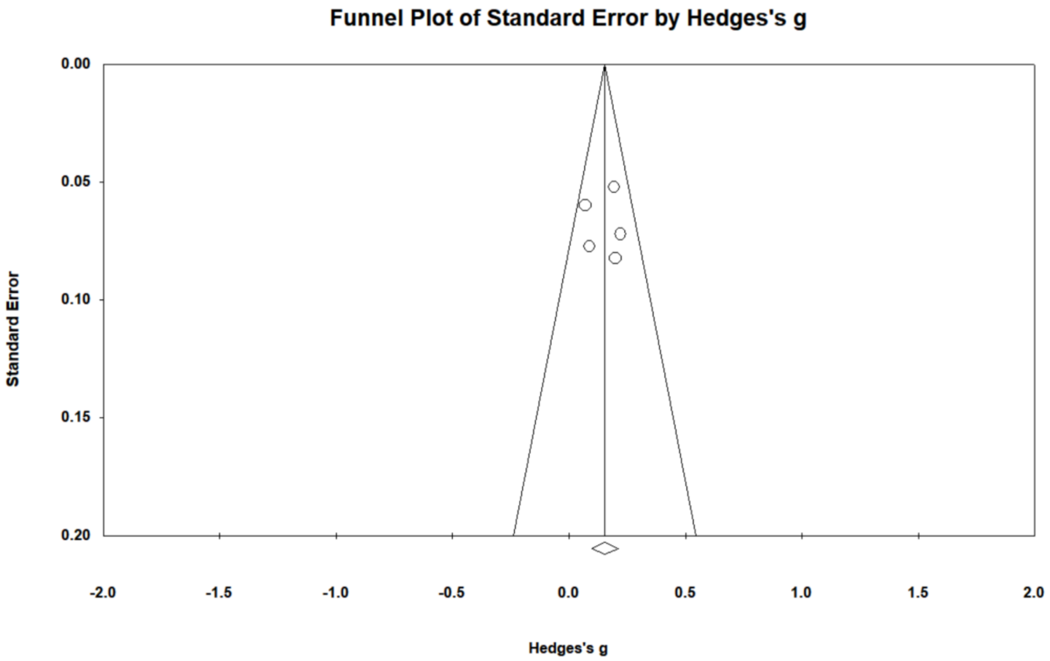

Figure SB8. DS & CN Group P-I funnel plot

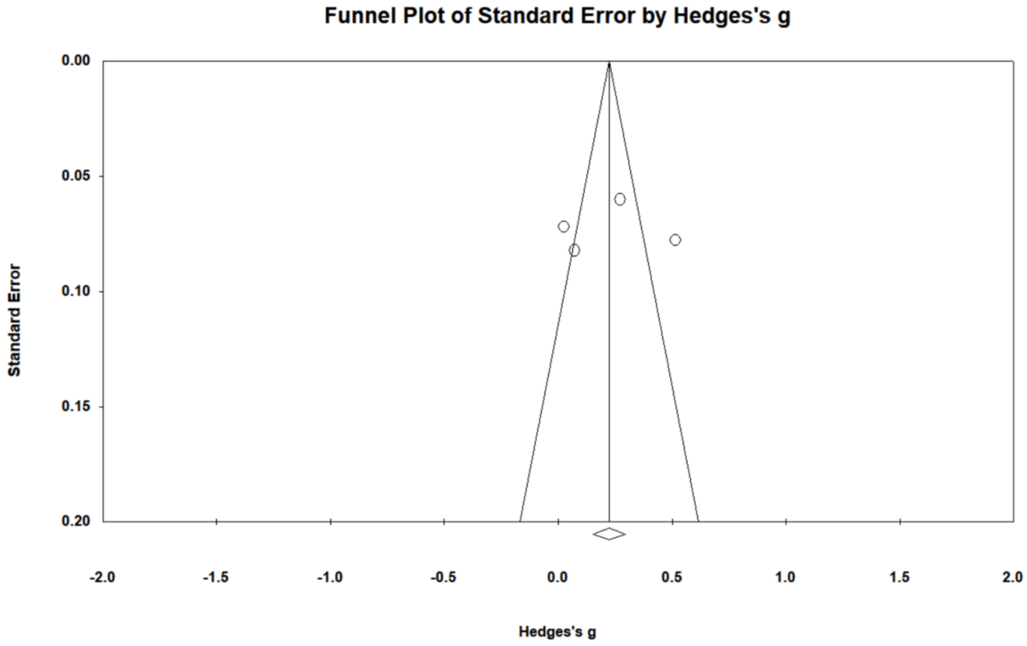

Figure SB9. DS & CN Group PSY funnel plot

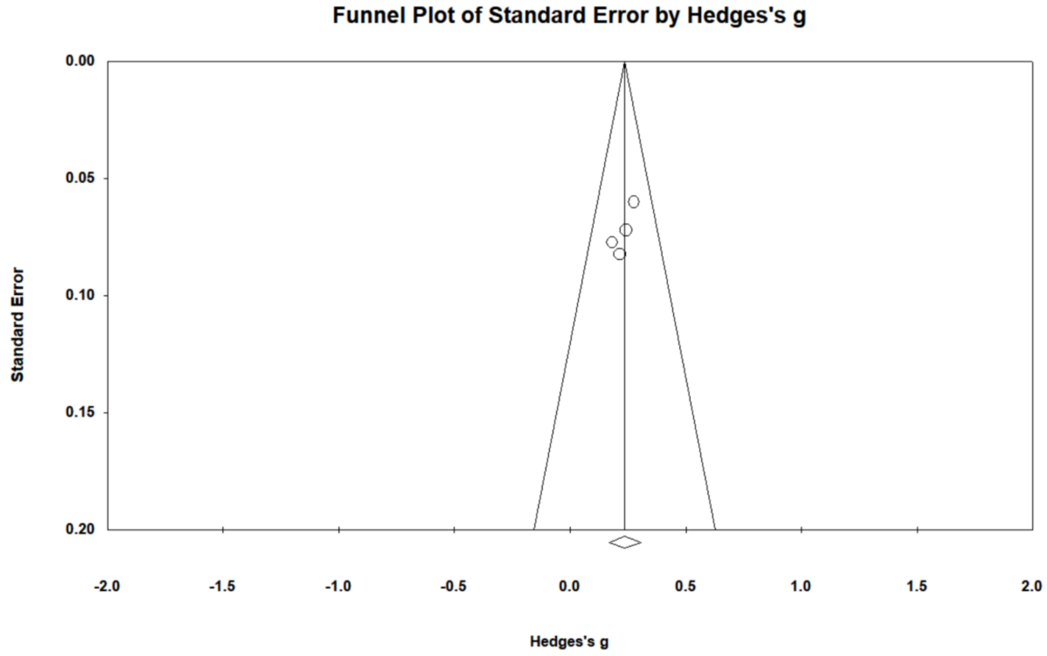

Figure SB10. DS & CN Group T-A funnel plot

Supplementary SC Funnel plots for Doctoral Students & Chinese Youth Norms Group

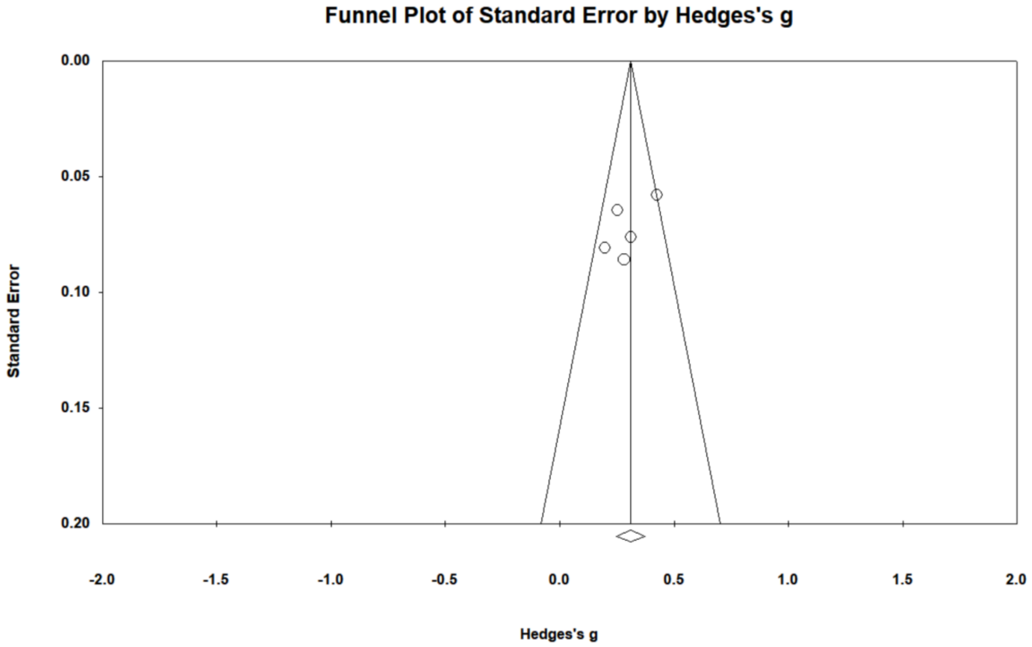

Figure SC1. DS & CYN Group SOM funnel plot

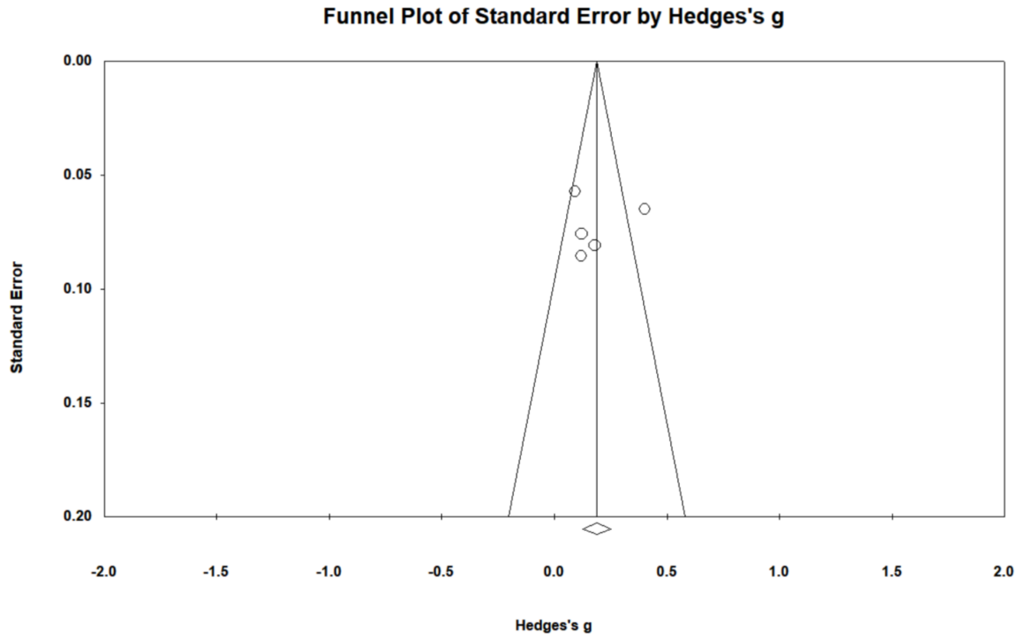

Figure SC2. DS & CYN Group O-C funnel plot

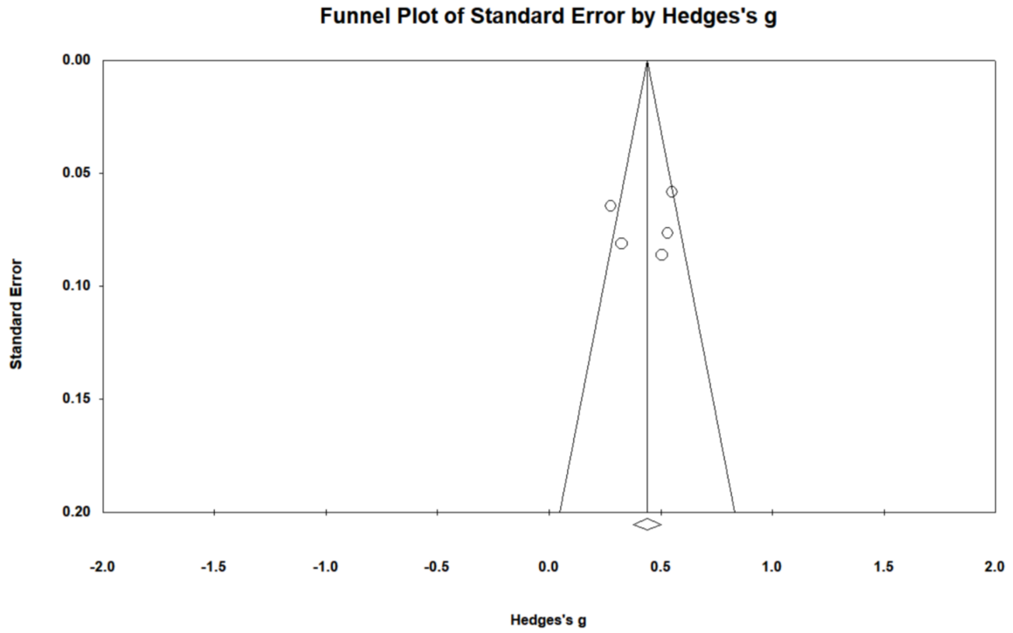

Figure SC3. DS & CYN Group I-S funnel plot

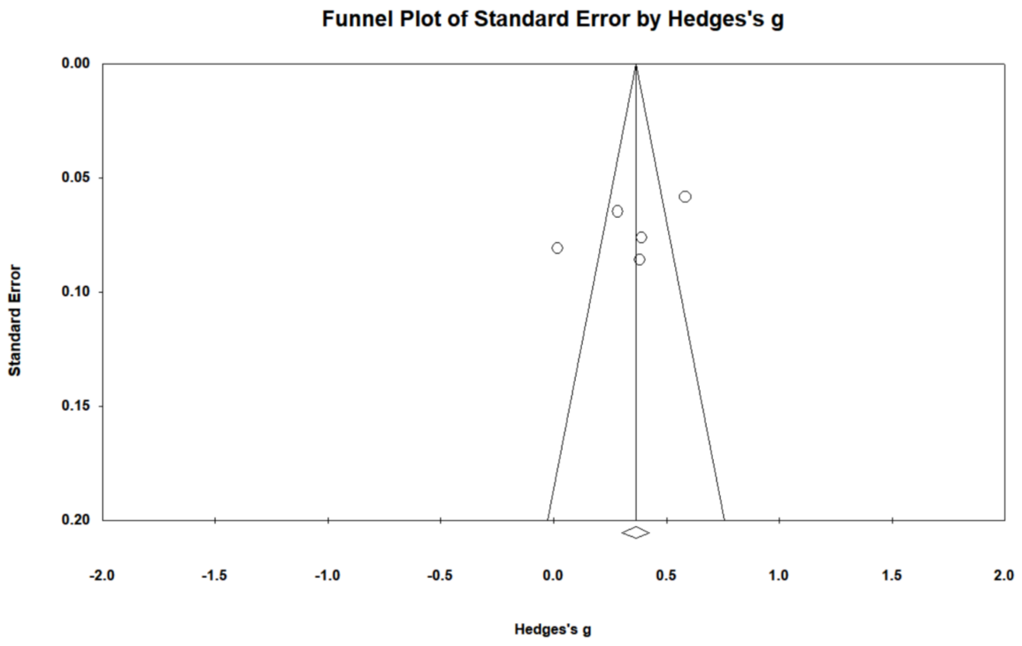

Figure SC4. DS & CYN Group DEP funnel plot

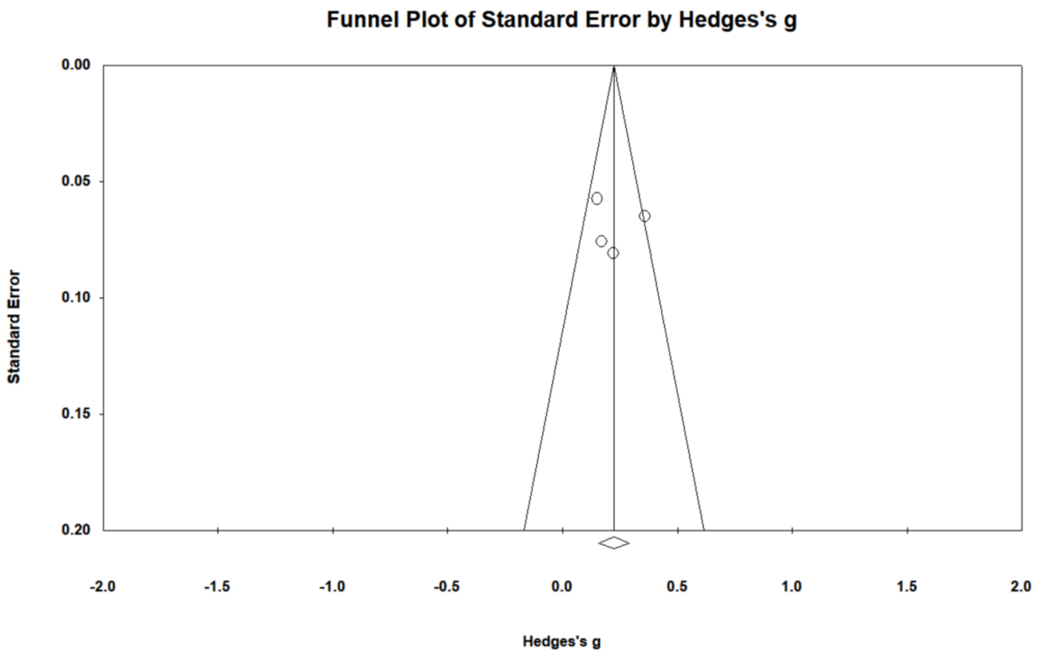

Figure SC5. DS & CYN Group ANX funnel plot

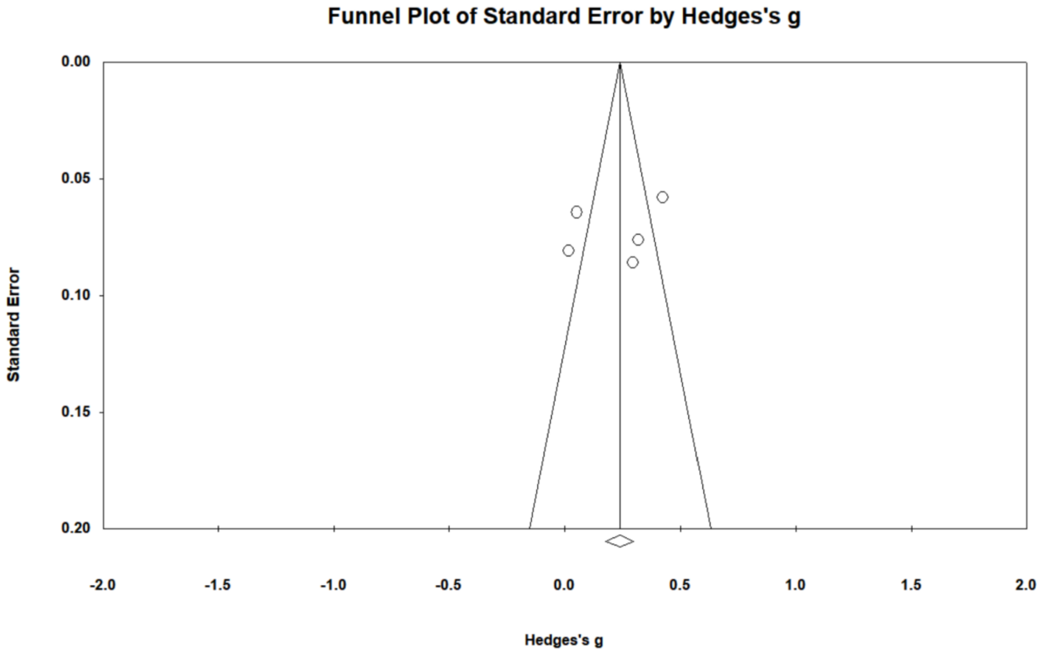

Figure SC6. DS & CYN Group HOS funnel plot

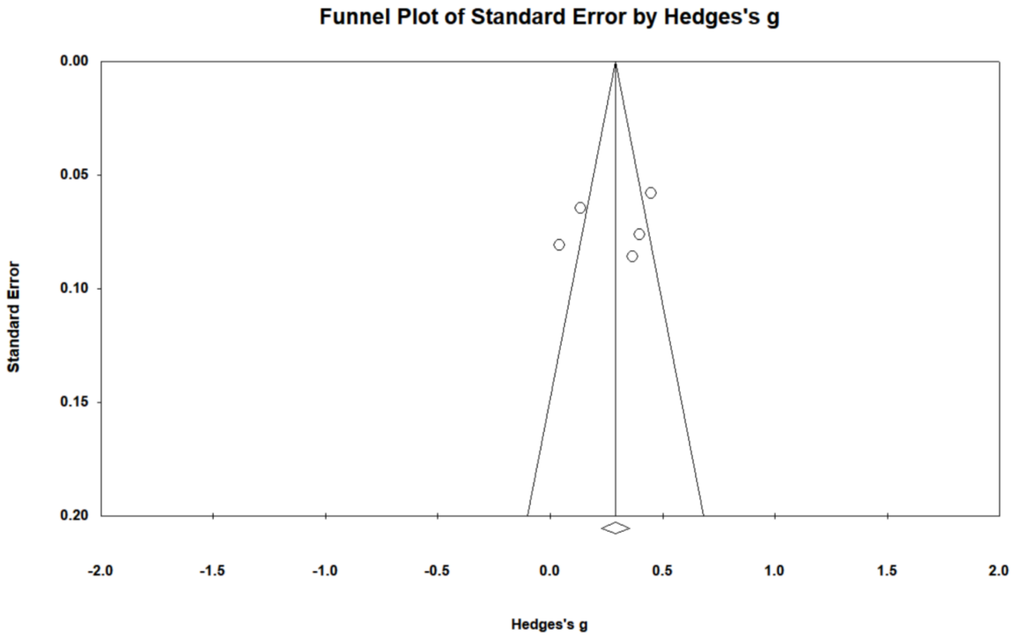

Figure SC7. DS & CYN Group P-A funnel plot

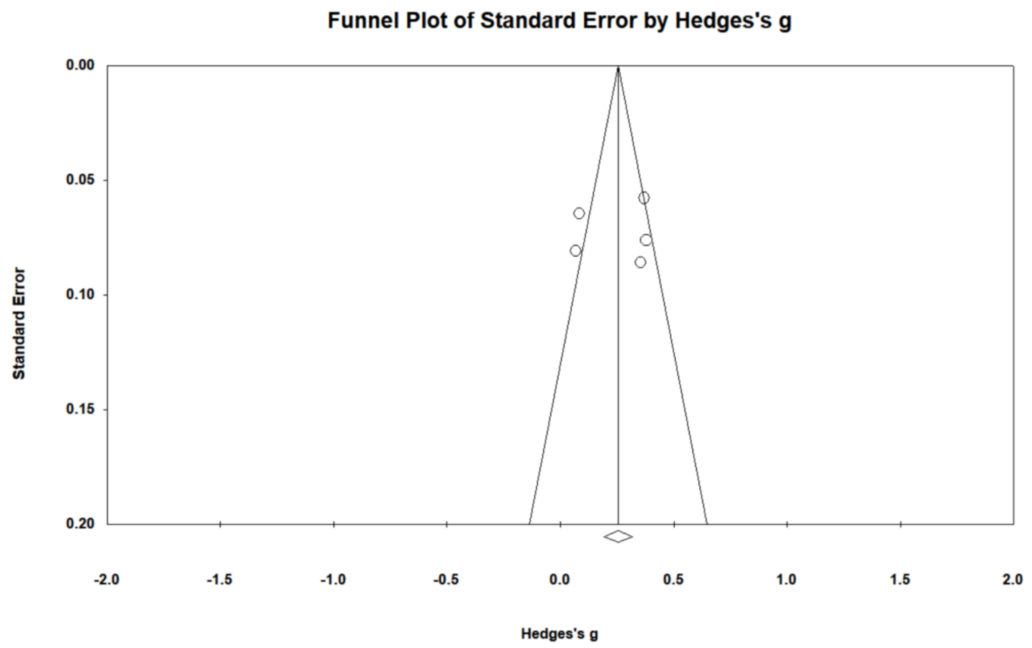

Figure SC8. DS & CYN Group P-I funnel plot

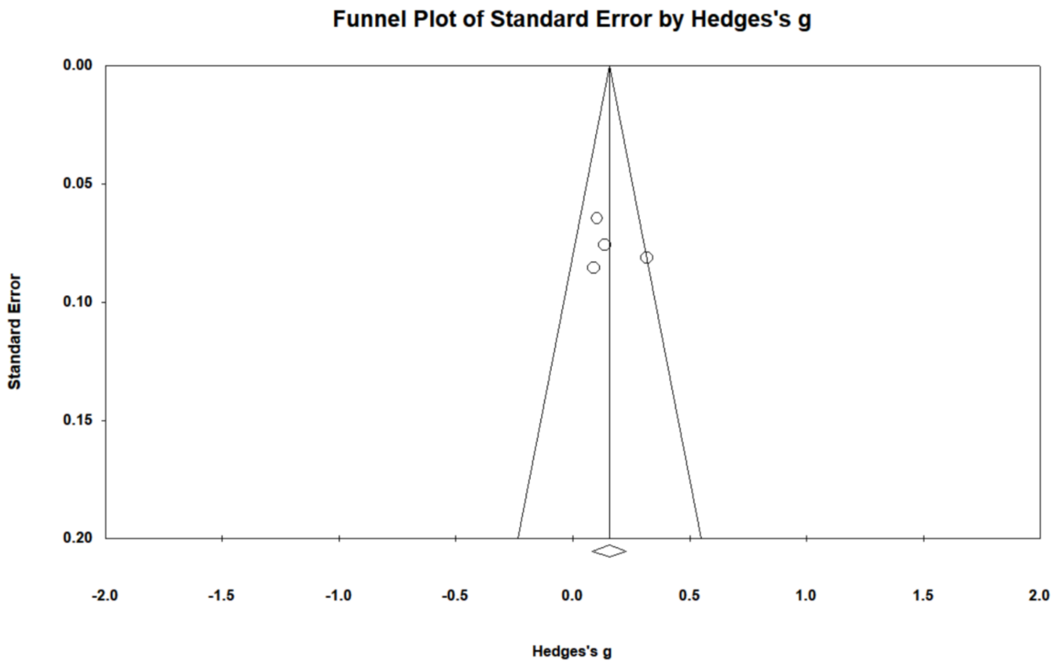

Figure SC9. DS & CYN Group PSY funnel plot
